# Temporal Phenotypic Changes in Huntington’s Disease Models for Preclinical Studies

**DOI:** 10.3233/JHD-210515

**Published:** 2022-03-01

**Authors:** Sophie St-Cyr, Alicia R. Smith, Beverly L. Davidson

**Affiliations:** a The Raymond G. Perelman Center for Cellular and Molecular Therapeutics, The Children’s Hospital of Philadelphia, Philadelphia, PA, USA; b The Department of Pathology & Laboratory Medicine, The Perelman School of Medicine, The University of Pennsylvania, Philadelphia, PA, USA

**Keywords:** Huntington’s disease, rotarod test, motor activity, muscle strength, narrow beam, descending rod

## Abstract

**Background:**

Mouse models bearing genetic disease mutations are instrumental in the development of therapies for genetic disorders. Huntington’s disease (HD) is a late-onset lethal dominant genetic disorder due to a CAG repeat within exon 1 of the Huntingtin (*Htt*) gene. Several mice were developed to model HD through the expression of a transgenic fragment (exon 1 of the human *HTT*), the knock-in mutation of the CAG repeat in the context of the mouse *Htt* gene, or the full-length *HTT* human gene. The different mouse models present distinct onset, symptoms, and progression of the disease.

**Objective:**

The objective of this study is to advise on the best behavioral tests to assess disease progression in three HD mouse models.

**Methods:**

We tested N171-82Q transgenic mice, zQ175 knock-in mice, and BACHD full-length mice in a comprehensive behavior test battery in early, mid-, and late disease stages.

**Results:**

We contrast and compare the models and the emerging phenotypes with the available literature. These results suggest the most effective behavioral tests and appropriate sample sizes to detect treatment efficacy in each model at the different ages. We provide options for early detection of motor deficits while minimizing testing time and training.

**Conclusion:**

This information will inform researchers in the HD field as to which mouse model, tests and sample sizes can accurately and sensitively detect treatment efficacy in preclinical HD research.

## INTRODUCTION

Huntington’s disease (HD) is a fatal autosomal dominant neurodegenerative disease characterized by CAG repeat expansion in exon 1 of *Huntingtin* (*HTT*) [1]. Mutant Huntingtin (mHTT) is ubiquitously expressed, and causes molecular dysfunctions and aggregation within cells [2]. Additionally, RAN translation, RNA toxicity and splicing dysregulation has been observed [3–5]. HD symptoms are generally detectable in the fourth decade of life and include cognitive decline, progressive involuntary movements (chorea), behavioral abnormalities, muscle wasting, and death within 10 to 20 years of symptom onset [6]. The prevalence of HD in Europe and the United States is 5–7 per 100,000, making HD among the more common inherited neurodegenerative diseases [7]. Despite mHTT’s ubiquitous expression and broad pathogenic effects, degeneration happens prominently in the striatum’s medium spiny neurons, constituting 90–95% of its neuronal population, followed by the cortex [8]. As the striatum is required for learning and performance of motor function [9], motor dysfunctions are among the earliest symptoms in HD patients [7]. Currently, only symptomatic treatment for HD is available, and recent clinical trials using gene silencing technologies with antisense oligonucleotides (ASO) were terminated as they presented a lack of target engagement or worsening of disease readouts [10, 11]. Thus, as the field advances, new ASO approaches, or other innovative methodologies require robust preclinical testing in mouse models.

Decades ago, animal models of HD were generated via targeted lesions or neurotoxin injuries to the striatum to create HD-like pathologies [12, 13]. The major limitation of these models was the lack of a progressive phenotype. The discovery of the *HTT* repeat expansion as the genetic cause of HD allowed the development of transgenic HD mouse models harboring varying exons of mHTT, knock-ins of expanded CAG-repeats (either pure CAG or with alternate codons) or full length transgenic models ([14–21] see [22] for review). In addition, conditional, cell- type or tissue-specific (astrocytes, hypothalamic, cortex pyramidal neurons, heart) models have been developed, along with those having distinct protein domain deletions [23–28]. A fully humanized model has also been developed which expresses the human *HTT* in the absence of endogenous murine *Htt* [29]. Longitudinal analysis of mutant phenotypes is commonly done and is necessary to identify and validate primary treatment endpoints. In all models, heterozygous mice more closely represent the patient genetic condition with varying onset.

An N-terminal polyglutamine fragment of mHTT is sufficient to elicit pathology and induce cytotoxicity [14]. Transgenic fragment models expressing exon 1 of *HTT* (or more) with a CAG expansion include the R6/1, R6/2 and N171-82Q models [14–16]. N171-82Q mice, used here, express *HTT* exons 1–3 from a prion protein promoter with a stable 82 glutamine repeat contrary to R6/2 which express a longer unstable repeat from its endogenous promoter [15]. The N171-82Q mouse model is a fast-progressing model showing weight loss at 8 weeks, motor deficits at 10 weeks and a shortened lifespan of ∼ 24 weeks [14, 30–32]. This model is suitable in short-term studies, with the limitation that in most instances N171-82Q males are used only, without a clear justification [32–35].

On the contrary, the poly(Q) knock-in models at the *Htt* locus, such as HttQ72-81, Htt^(CAG) 150^ and zQ175 mice, have a slower onset and a more gradual phenotype with no decrease in longevity [17–19]. The full-length knock-in models recapitulate more faithfully the human mutation in the murine genetic context than the transgenic models. Here, we use zQ175 mice as representative of the knock-in models, which express around 190 (range 180–220) CAG repeats within the human exon 1 under the human Huntingtin homolog (Htt) promoter. Because the repeat is pure CAGs, it is not stable [1]. Heterozygous mice manifest a mild phenotype with decreased weight at 6.5 months in males and 10 months in females, nocturnal behavioral deficits starting at 8 to 10 months, and variable increases in anxiety-like behavior through reduced motivation in reward trials [17, 36]. Often, homogeneous knock-in mice are used as they present with more rapid disease progression and a reduced lifespan [17, 37, 38].

Full-length mouse models expressing mHTT in a yeast or bacterial artificial chromosome include the BACHD and YAC128 models. These mice have 97 and 100–126 CAG repeats with transgene copy numbers of ∼5 and ∼4 respectively, and normal lifespan [20, 21]. We chose BACHD as representative of the full-length transgenic models. BACHD mice express a stable CAA-CAG mixed repeat under the control of the endogenous regulatory machinery [21], and have progressive motor deficits starting around 6 months with neurodegeneration, diffuse nuclear mHTT accumulation and increased anxiety-like behavior at 6 months [36, 39–41]. In BACHD mice, hypothalamic mHTT impairs glucose metabolism, which is associated with an increase in food intake and body weight gain starting at 2 months [25]. Food-restricted or weight-corrected BACHD performances indicate a robust rotarod performance deficit [42]. Although less appropriate for metabolic studies, these models constitute an important tool to test potential therapies aimed specifically at the human *HTT* sequences that are 3^′^ of those contained within transgenic fragment models.

Motor behavior assessment is one method to evaluate the efficacy of potential HD treatments and can be achieved through robust tests in regulated conditions [43]. Here, we exposed naïve N171-82Q, zQ175 and BACHD hemizygous mice of both sexes, in early, mid-, and late disease to an array of phenotypic tests. This battery of tests includes weight tracking, rotarod performance and learning, grip strength, descending rod, narrow beam, and activity chamber activity. Additionally, we assessed climbing behavior. Altogether, these assessments assess fine motor skills, balance, learning, coordination and general locomotor activity [44]. Each test is standardized and requires minimal or no training. Based on our data, sample sizes required to detect a therapeutic effect size of 25, 50 or 75% are provided for sexes combined or separate for each mouse model. Cumulatively, this report provides updated guidance to the community of researchers developing novel therapies for HD, including the opportunity to detect therapeutic benefits earlier in the disease course.

## MATERIALS AND METHODS

### Animals

Hemizygous N171-82Q (B6C3-Tg(HD82Gln)81Gschi/J) and zQ175 (B6J.129S1-*Htt^*tm*1*Mfc*^*/190ChdiJ) males were bred to C57BL/6 females. Hemizygous BACHD (FVB/N-Tg(HTT^*^97Q)IXwy/J) males were bred to FVB/N females. N171-82Q mice were genotyped using F 5^′^-ATG GCG ACC CTG GAA AAG CTG-3^′^ and R 5^′^-TCG GTG CAG CGG CTC CTC-3^′^ primers. zQ175 mice were genotyped using F 5^′^-AGA GCA GCC GAT TGT CTG TTG-3^′^ and R 5^′^-GAT CGG CCA TTG AAC AAG ATG-3^′^ primers. BACHD mice were genotyped using F 5^′^-ATG GCG ACC CTG GAA AAG CTG-3^′^ and R 5^′^-GGT CGG TGC AGA GGC TCC TC-3^′^ primers. Hemizygous animals were used as well as age-matched wild-type (WT) littermates. For each litter, a maximum of two animals per sex and genotype were used. Animals were housed on a 12-h light/dark cycle with light on at 6:15AM and *ad libitum* access to food and water in an enriched and temperature-controlled environment. The cage enrichments included a shelter and nesting material. The behavioral protocols complied and approved by the Animal Care and Use Committee at the Children’s Hospital of Philadelphia.

### Behavioral testing

**Fig. 1:**
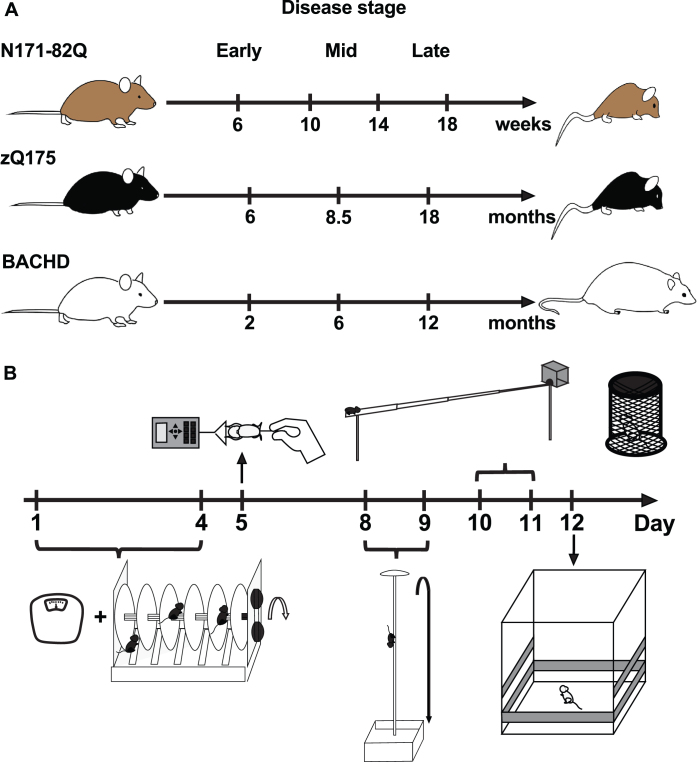
Study design. A) Progressive behavioral phenotype and testing times throughout the disease in three HD mouse models. B) Motor behavior test battery timeline. Tasks represented include, chronologically: weighing, accelerating rotarod, forelimb grip strength, descending rod, narrow beam, and activity chamber. The climbing test was conducted independently. The order of tasks is a suggestion.

Mice were weighed and habituated to the test room for at least one hour before any test. Each animal was used at one time-point only to avoid a carry-on effect of learning from previous behavioral tasks [45], therefore providing an accurate baseline and phenotype for any given age. Animals were tested in early (6 weeks in N171-82Q, 6 months in zQ175, 2 months in BACHD), mid- (10 weeks in N171-82Q, 8.5 months in zQ175, 6 months in BACHD) and late disease (14 and 18 weeks in N171-82Q, 18 months in zQ175, 12 months in BACHD; Fig. 1A). Tests were performed successively over a 12-day period in the following order: rotarod, forelimb grip strength, descending rod, narrow beam, and activity chamber. The climbing test was performed on a separate set of BACHD mice (Fig. 1B). Approximately 15 mice (5–23 range) per age, sex, and genotype were tested per group depending on their availability. Due to their initially smaller available sample size, a larger BACHD males sample size was tested independently and gave the same results as the smaller male group. zQ175 mice could not be tested for forelimb grip strength or on the descending rod at 8.5, 18, and 24 months and at any time on the narrow beam due to a limited access to the testing apparatus. Similarly, N171-82Q mice at 10, 14, and 18 weeks could not be tested on the narrow beam. Behavioral testing was conducted in the morning, at least an hour after light onset, to minimize circadian variation in activity.

### Accelerating rotarod

The accelerating rotarod (Ugo Basile, Comerio, Italy) test was carried out as previously described [46] on days 1 to 4 of the behavioral test battery (Fig. 1B). Briefly, on the first day, mice were trained for 5 min at 5 rpm and then tested in three trials per day, with at least 30 min between trials for four consecutive days. In each trial, the rotarod accelerated from 5 to 40 rpm over 4 min with a constant speed of 40 rpm for an additional minute. Trials were stopped at 300 s. Latency to fall (or two consecutive rotations without running) was recorded for every trial. Data for each mouse, model and time point were analyzed using a 2-way ANOVA with genotype and testing day as main effects. A Sidak’s or Dunnett’s multiple comparison test was done to evaluate performance and learning (improvement over testing days) of the task within and between the genotypes with sexes combined or separated.

### Forelimb grip strength

Forelimb grip strength took place on day 5 of the behavioral test battery. Peak tension was measured five times per mouse (Columbus Instrument, USA) with an inter-trial time of at least 30 min. The mouse held a pull bar with both paws and were then pulled horizontally until they let go of the bar, producing a reading of the maximum strength required to hold onto the bar in gram of force (Fig. 1B). The grip strength is calculated as the average of the four highest grip strengths recorded. Data for each model and time point were analyzed using a Student’s *t*-test with or without Welch’s correction or a Mann-Whitney test to compare genotypes with sexes combined or separated.

### Descending rod

Mice were tested on the descending rod on day 8 and 9 of the behavioral test battery (Fig. 1B). The descending rod was 15 mm in diameter and 80 cm long. The rod was placed in a mouse cage with bedding. Animals were placed at the top of the rod facing upwards and the time to start the descent (latency to descend), to turn perpendicular to the ground (T-turn) and reach the bottom of the rod (T-total) were measured. Five trials were done with at least 30 min between trials on two consecutive days. The first day constitutes training while the second is the test day. The behavior was filmed and scored by an observer who was blinded to genotype. The latency, T-turn, and T-total were calculated as the average of the four shortest times recorded. Late disease (18 weeks) N171-82Q female mice were unable to learn this task. Data for each model and time point were analyzed using a Student’s *t*-test with or without Welch’s correction or a Mann-Whitney test to compare genotypes with sexes combined or separated.

### Narrow beam

Mice were tested on the narrow beam on day 10 and 11 of the behavioral test battery (Fig. 1B). The narrow beam was made of 4 consecutive clear Plexiglass sections of decreasing width (30 mm, 20 mm, 15 mm, and 10 mm) of 25 cm length each, totaling one meter in distance to reach an enclosed safety platform. The narrow beam is increasingly elevated from 40 to 47 cm as mice present a natural tendency to climb upward to escape uncomfortable situations. The latency to start crossing the beam, the time to cross and the number of slips were recorded. Mice crossed the beam 5 times with at least 30 min between the trials on two consecutive days. The first day constitutes training while the second is the test day. The behavior was filmed and measured by an observer blinded to genotype. The average of the four shortest crosses was calculated. Data for each model and time point were analyzed using a Student’s *t*-test with or without Welch’s correction or a Mann-Whitney test to compare genotypes with sexes combined or separated.

### Climbing test

Groups of 2-, 4-, and 12-months old BACHD mice were tested (Fig. 1B). The climbing test consists of a metal wire mesh pencil holder (11 cm diam. X 17 cm heigh) in which the mouse is placed for 5 min while being video recorded. The frequency and duration of vertical activity (rearing, rearing while leaning on the side and climbing) is recorded by an observer blinded to genotype. Data for each time point were analyzed using a Student’s *t*-test with or without Welch’s correction or a Mann-Whitney test to compare genotypes with sexes combined or separated.

### Activity chamber

Mice were tested in the activity chamber on day 12 of the behavioral test battery (Fig. 1B). An activity chamber apparatus (28 cm×28 cm×20 cm; Medicine Associates Inc., USA) using infrared beam crossing to measure activity was used. The mouse activity recorded included the distance travelled, the jumping frequency and the rearing time over 30 min (N171-82Q), 20 min (zQ175), or 60 min (BACHD). Total distance travelled, rearing time, and jump frequency and for each model and time point were analyzed using a Student’s *t*-test with or without Welch’s correction or a Mann-Whitney test to compare genotypes with sexes combined or separated. Data separated into 10-min time bins were analyzed using a 2-way ANOVA with the genotype and time bin as the main factors with Sidak’s or Dunnett’s multiple comparison tests within and between the genotypes with sexes combined or separated.

### Statistics

Analyses were conducted using Prism 8. Normality of the distributions was assessed by the Shapiro-Wilk test. Effect sizes (Cohen’s d; https://www.socscistatistics.com, https://www.campbellcollaboration.org) [47] and sample sizes required to detect a 25%, 50% or 75% therapeutic benefit (alpha 0.05, 80% power; https://epitools.ausvet.com.au) [48, 49] were calculated for the behavioral tests showing statistical significance.

We report results with medium to large effect sizes only. Analyses were conducted by comparing each HD mouse model to age-matched WT mice of their strain background. Further, both sexes were analyzed together and then separately to detect potential sex effects. Outliers (> ±2 standard deviations) were removed from groups when sexes were pooled. Weight correction for the mouse model was done by removing the HD animals outside two standard deviations of the WT mice weight or by dividing the mouse performance by its weight. Overall, N171-82Q motor performances were not affected by weight. On the contrary, zQ175 and BACHD mice forelimb grip strength and BACHD jump frequency in the activity chamber are influenced by the weight of the animal.

## RESULTS

### Weight

Weight is a non-invasive and important indicator of the general health, physiological and metabolic state in mouse models of human neurological disease. Variation in weight is explained in part by muscle wasting in patients and animal models of HD in conjunction with a decrease in the conversion of calories into mass [50, 51] and/or the expression of the disease allele especially in the hypothalamus [25]. Historically, N171-82Q and zQ175 mice weigh less starting at approximately 8 weeks and 12 months respectively [14, 52] while BACHD mice are heavier starting early in the disease (2 months) [25].

**Fig. 2:**
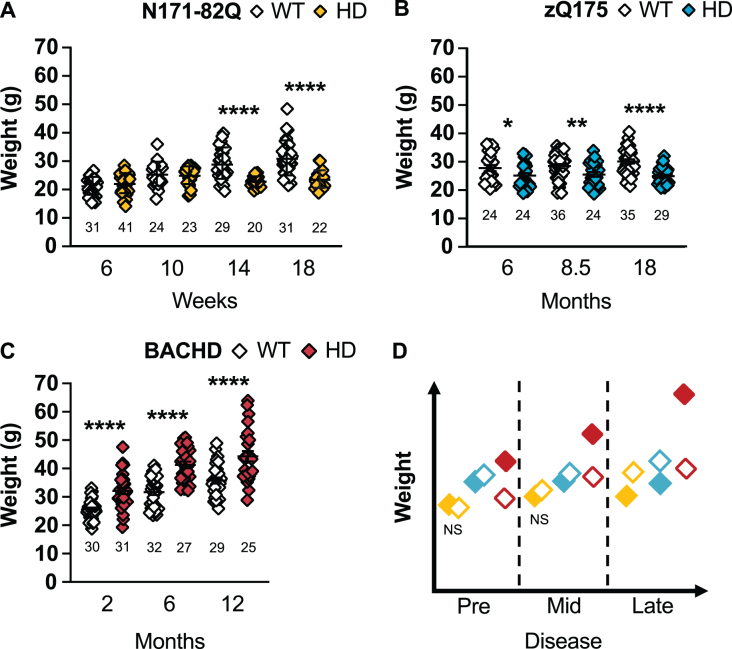
HD mice differ in weight throughout the disease. N171-82Q mice weigh less in late disease (14 weeks: *t* = 4.51, *df* = 47, *p* < 0.0001; 18 weeks: *t* = 5.730, *df* = 47, *p* < 0.0001) (A) while zQ175 mice always weigh significantly less (6 months: *MWU* = 199.5, *p* = 0.03; 8.5 months: *t* = 2.548, *p* = 0.007; 18 months: *t_*w*_* = 5.492, *p* < 0.0001) than WT mice (B). BACHD mice weigh more than WT mice throughout the disease (2 months: *t_*w*_* = 5.223, *p* < 0.0001; 6 months: *t* = 6.499, *p* < 0.0001; 12 months: *t* = 4.163, *p* < 0.0001) (C). D) Representative model of weight variation in the three mouse models as the disease progresses. All comparisons are made to WT mice of the same background. Sample sizes are presented under each group. HD, Huntington’s disease carrier; NS, non-significant; WT, wild-type. Data represent mean ± SEM. ^*^p ≤ 0.05, ^*^p ≤ 0.01, and ^****^p ≤ 0.0001 indicate a significant difference by a Student’s *t*-test with or without Welch’s correction or Mann Whitney test for each time point.

N171-82Q mice present a significant weight loss in mid- to late disease (14 and 18 weeks: *p* < 0.0001; Fig. 2A). This weight loss starts at 10 weeks in males (10 weeks: *p* = 0.04, 14 and 18 weeks: *p* < 0.0001; Supplementary Figure 1A). On the contrary, N171-82Q females are initially heavier at mid-disease (10 weeks: *p* = 0.03) and fail to gain weight by late disease (14 weeks: *p* = 0.03, 18 weeks: *p* = 0.0002; Supplementary Figure 1B). Sample sizes of 8 to 13 mice in mixed-sex groups are necessary to detect a 50% weight rescue with males requiring smaller (6–9) and females (13) slightly larger sample sizes to detect the same difference (Table 1, Supplementary Tables 3 and 4).

**Table 1:** Recommended sample sizes to detect a therapeutic benefit for different behavioral tests in three HD mouse models

Mouse model	N171-82Q				zQ175			BACHD		
Disease stage	Early	Mid	Late	Late	Early	Mid	Late	Early	Mid	Late
Age (Unit)	6	10	14	18	6	8.5	18	2	6	12
	Weeks				Months			Months		
Weight									
25%	ND	ND	21 / grp	35 / grp	ND	ND	ND	ND	ND	ND
50%			**<6 / grp**	**9 / grp**	ND	ND	**16 / grp**	42 / grp	**20 / grp**	58 /grp
75%			**<6 / grp**	**<6 / grp**	62 / grp	46 / grp	**8 / grp**	**19 / grp**	**9 / grp**	26 /grp
Other					100% : 35 / grp	100% : 26 / grp		**85% : 15 / grp**		**100% : 15 / grp**
Rotarod Performance									
25%	ND	ND	ND	**<6 / grp**	ND	ND	ND	ND	54 / grp	ND
50%	ND	ND	ND	**<6 / grp**		ND	ND	27 / grp	**15 /grp**	ND
75%	ND	49 / grp	**20 / grp**	**<6 / grp**		44 / grp	ND	**12 / grp**	**7 /grp**	53 /grp
Other	100% : 50 / grp	100% : 28 / grp	**100% : 12 / grp**			100% : 25 / grp	100% : 47 / grp			100% : 30 / grp
FGS									
25%	ND	ND	ND	**10 / grp**	ND	NA	NA	56 / grp*	40 / grp*	ND
50%	54 / grp	21 / grp	31 / grp	**<6 / grp**	48 / grp			15 / grp*	10 / grp*	43 / grp*
75%	24 / grp	**10 / grp**	**14 / grp**	**<6 / grp**	22 / grp			7 / grp*	5 / grp*	19 / grp*
Other	**95% : 15 / grp**				**90% : 15 / grp**					100% : 11 / grp*
DR – T-turn									
25%	ND	ND	ND	ND	ND	NA	NA	58 / grp	ND	23 / grp
50%		ND						**15 / grp**		**6 / grp**
75%		43 / grp						**7 / grp**		**<6 / grp**
Other		100% : 24 / grp							
DR – T-total									
25%	ND	ND	ND	ND	ND	NA	NA	ND	ND	**18 / grp**
50%	ND	45 / grp			ND			**<6 / grp**	27 / grp	**<6 /grp**
75%	31 / grp	**20 / grp**			32 /grp			**<6 / grp**	**12 /grp**	**<6 /grp**
Other	**100% : 18 /grp**	**90% : 14 / grp**			**100% : 18 /grp**				
DR – Latency									
25%	**<6 / grp**	ND	ND	ND	ND	NA	NA	**<6 / grp**	ND	ND
50%	**<6 / grp**		48 / grp		ND			**<6 / grp**	
75%	**<6 / grp**		22 / grp		ND			**<6 / grp**	
Other			**90% : 15 /grp**		100% : 43 /grp				
NB – Cross time									
25%	52 / grp	NA	NA	NA	NA	NA	NA	**12 / grp**	54 / grp	ND
50%	23 / grp							**<6 / grp**	**14 / grp**	**20 / grp**
75%	**15 / grp**							**<6 / grp**	**6 / grp**	**9 / grp**
Other									
NB – Slips									
25%	ND	NA	NA	NA	NA	NA	NA	ND	ND	ND
50%								ND	ND	ND
75%								35 / grp	ND	42 / grp
Other								**100% : 20 / grp**	100% : 36 / grp	100% : 24 / grp
NB – Latency									
25%	ND	NA	NA	NA	NA	NA	NA	ND	ND	ND
50%	62 / grp									ND
75%	28 / grp									57 / grp
Other	**100% : 16 /grp**									100% : 37 / grp
CT – Frequency									
25%	NA	NA	NA	NA	NA	NA	NA	ND	**4 months old**	ND
50%								41 / grp*	ND	19 / grp*
75%								19 / grp*	42 / grp*	9 / grp*
Other								100% : 11 / grp*	100% : 24 / grp*
AC – Distance									
25%	ND	ND	ND	ND	ND	ND	ND	ND	ND	33 / grp
50%							22 / grp	35 / grp		**9 / grp**
75%							**10 / grp**	**16 / grp**		**<6 / grp**
Other								**80% : 14 / grp**	
AC – Jump									
25%	ND	ND	ND	ND	ND	ND	ND	ND	29 / grp	**16 / grp**
50%				42 / grp				ND	8 / grp*	**<6 / grp**
75%				**19 / grp**				6 / grp*	<6 / grp*	**<6 / grp**
Other				**85% : 15 / grp**				100% :<6 / grp*	
AC – Rearing									
25%	ND	ND	ND	ND	ND	ND	ND	ND	ND	ND
50%	ND			ND			ND			22 / grp
75%	55 / grp			37 / grp			42 / grp			**10 / grp**
Other	100% : 45 /grp			100% : 21 / grp			100% : 24 / grp		

HD mice recommended sample sizes to detect a normalization of the behavior by 25, 50, 75% or more. Sample sizes in bold are practical. AC, Activity chamber; CT, Climbing test; DR, Descending rod; FGS, Forelimb grip strength; NA, Not available; NB, Narrow beam; ND, Not determined; T-time, Time to descend the descending rod; T-turn, Time to turn downward on the descending rod; ^*^Weight-corrected data.

zQ175 mice are significantly lighter at all stages of disease (6 months: *p* = 0.03, 8.5 months: *p* = 0.007, 18 months: *p* < 0.0001; Fig. 2B). Similarly, zQ175 males and females separately are lighter at all time points investigated (male –6 months: *p* = 0.006, 8.5 months: *p* = 0.02, 18 months: *p* < 0.0001; females: 6 months: *p* = 0.04, 8.5 months: *p* = 0.007, 18 months: *p* = 0.0001, 24 months: *p* = 0.006; Supplementary Figure 1C, D). Eleven females are necessary to detect a 75% treatment-related improvement in weight in mid-disease while 9 males or females and 16 mice of mixed-sexes are required to detect a 50% treatment-related improvement in weight in late disease.

Contrary to N171-82Q and zQ175 mice, BACHD mice are heavier at all time points (*p* < 0.0001; Fig. 2C). BACHD males are heavier in early and mid-disease stages (2 months: *p* = 0.0007, 6 months: *p* = 0.003; Supplementary Figure 1E) and BACHD females at all stages (2 months: *p* = 0.0003, 6 and 12 months: *p* < 0.0001; Supplementary Figure 1F). Larger sample sizes (> 20 mice) are required to detect a 50% -related treatment rescue and 20 males, or 12 mice of mixed-sex, are required to detect a 75% treatment-related improvement in weight in early or mid-disease. Less mice (7-19) are required to detect female’s weight correction throughout the disease.

### Accelerating rotarod

The accelerating rotarod is one of the ‘gold standard’ behavioral tasks to evaluate motor performance (gait and inter-limb coordination), strength, endurance, balance and learning through the corticostriatal pathway [53–55], including in HD rodent models [56] (Fig. 1B). In HD animals, time spent on the rotarod declines with disease progression [57]. Historically, the three models perform worse on the rotarod starting as early as 10 weeks, 12 months, and 4 weeks of age for N171-82Q, zQ175, and BACHD mice, respectively [31, 58, 59]. A lack of motivation, can artificially reduce the latency to fall and these data should be excluded from the analysis [60]. That lack of motivation can be detected through the lack of engagement in the task and a premature fall from the rod compared to the other mice from the group. Weight is also a confounding factor as heavier mice typically perform more poorly than lighter mice [45].

### N171-82Q

**Fig. 3:**
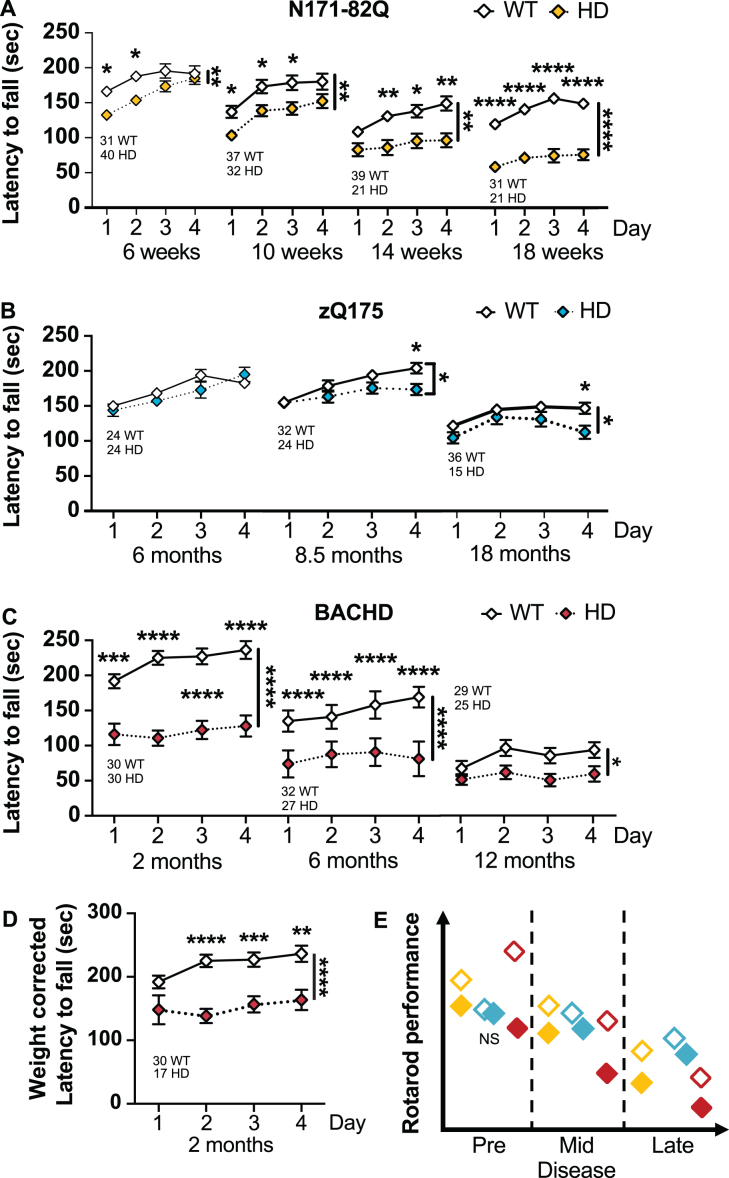
HD mice perform poorly on the accelerating rotarod. A) N171-82Q mice fall earlier from the rotarod throughout the disease (6 weeks: Genotype: *F_(1,69)_* = 7.430, *p* = 0.008, Time: *F_(2,151)_* = 17.99, *p* < 0.0001, *Post-hoc* Day 1-2: *t* = 2.951, *p* = 0.01; 10 weeks: Genotype: *F_(1,67)_* = 7.644, *p* = 0.007, Time: *F_(2,129)_* = 35.84, *p* < 0.0001, *Post-hoc* Day 1-3: *t* = 2.654, *p* = 0.04; 14 weeks: Genotype: *F_(1,58)_* = 11.03, *p* = 0.002, Time: *F_(2,132)_* = 14.96, *p* < 0.0001, *Post-hoc* Day 2–4: *t* = 3.085, *p* = 0.01; 18 weeks: Genotype: *F_(1,50)_* = 43.89, *p* < 0.0001, Time: *F_(3,141)_* = 14.79, *p* < 0.0001, *Post-hoc* Day 1-4: *t* = 5.790, *df* = 50, *p* < 0.0001, *d* = 1.6). B) zQ175 mice fall earlier from the accelerating rotarod starting in mid-disease (8.5 months: Genotype*Time: *F_(3,162)_* = 3.661, *p* < 0.001, *Post-hoc* Day 2-4: *t* = 2.728, *p* = 0.03; 18 months: Genotype: *F_(1,49)_* = 3.765, *p* = 0.05, Time: *F_(3,131)_* = 12.76, *p* < 0.0001, Post-hoc Day 4: *t* = 2.767, *p* = 0.04). C) BACHD mice fall earlier from the rotarod throughout the disease (2 months: Genotype: *F_(1,58)_* = 42.40, *p* < 0.0001, Time: *F_(2,141)_* = 6.258, *p* = 0.001, *Post-hoc* Day 1-4: *t* = 4.150, *p* = 0.0005; 6 months: Genotype: *F_(1,57)_* = 43.26, *p* < 0.0001, Time: *F_(3,171)_* = 9.597, *p* < 0.0001, *Post-hoc* Day 1-4: *t* = 5.304, *p* < 0.0001; 12 months: Genotype: *F_(1,52)_* = 5.055, *p* = 0.03). D) The difference in BACHD performance on the rotarod in early disease remains significant after weight correction (Genotype: *F_(1,45)_* = 17.83, *p* = 0.0001, Time: *F_(2,104)_* = 4.807, *p* = 0.007, *Post-hoc* Day 2-4: *t* = 3.555, *p* = 0.004). E) Representative model of accelerated rotarod performance variation in the HD mouse models as the disease progresses. All comparisons are made to WT mice of the same background. Sample sizes are presented under each group. HD, Huntington’s disease carrier; NS, non-significant; WT, wild-type. Data represent mean ± SEM. ^*^p ≤ 0.05, ^**^p ≤ 0.01, ^***^p ≤ 0.001, and ^****^p ≤ 0.0001 indicate a significant difference by a 2-way repeated measure ANOVA with Sidak’s multiple comparison tests for each time point. Side bars indicate a genotype effect. Brackets indicate a genotype and time interaction.

N171-82Q mice in early and mid-disease (6 weeks) perform more poorly on the accelerating rotarod compared to WT mice (6 weeks: genotype: *p* = 0.008, 10 weeks: genotype: *p* = 0.007, Fig. 3A). N171-82Q males are mainly responsible for this phenotype in early disease (genotype: *p* = 0.009; Supplementary Figure 2A) while females are responsible for the mid-disease detected decrease in performance (genotype: *p* = 0.006; Supplementary Figure 2B). At these disease stages, N171-82Q mice learn and improve at the task through repetition (Supplementary Figure 3A, 4A, 4B).

In late-disease, N171-82Q mice present a further decline in performance (14 weeks –genotype: *p* = 0.002; 18 weeks –genotype: *p* < 0.0001). Further, N171-82Q mice show learning deficits with no improvements over time, unlike WT mice (14 and 18 weeks –time: *p* < 0.0001). N171-82Q males and females individually show impaired performance on the rotarod at these time points (males –14 weeks genotype: *p* = 0.0007; 18 weeks genotype: *p* < 0.0001; females –18 weeks genotype: *p* = 0.001). Further, both males and females individually present impaired learning compared to WT mice (male 14 weeks –time: *p* < 0.0001; female 14 weeks –time^*^genotype: *p* = 0.03). Changes in late disease are robust and require sample sizes of less than 6 mice per genotype to detect a 50% improvement, including in male- and female-only groups.

### zQ175

zQ175 mice perform poorly on the accelerated rotarod when compared with WT mice in mid- and late-disease (8.5 months –time*genotype: *p* < 0.01; 18 months –genotype: *p* = 0.05; Fig. 3B). Similarly, zQ175 males perform poorly on the accelerated rotarod in mid-disease (8.5 months –time*genotype: *p* = 0.03; Supplementary Figure 2C). Females performed poorly in late disease (24 months –genotype: *p* = 0.02; Supplementary Figure 2D). These changes are detectable due to the small variance within groups. zQ175 mice of both genotypes and sexes learned the task similarly over time at all disease stages, except late-disease zQ175 females who surprisingly improve slightly their performance over time (18 months –time: *p* = 0.006; Supplementary Figure 3B, 4C, 4D). At mid-disease, 17 male mice are necessary to detect a 75% improvement and 15 females are necessary in late disease to detect a 50% improvement in performance. However, late disease performance is partly dependent upon the weight loss of the animal.

### BACHD

Early and mid-symptomatic BACHD mice perform poorly on the accelerated rotarod when com-pared to WT mice (genotype: *p* < 0.0001), including when the performance is corrected for body weight (genotype: *p* = 0.0001; Fig. 3C). Similarly, BACHD males and females perform poorly on the rotarod at these stages (male –genotype 2 months: *p* < 0.0001, 6 months: *p* = 0.0002; female –genotype 2 months: *p* = 0.0002, 6 months: *p* < 0.0001; Supplementary Figure 2E, F). BACHD mice also fail to learn relative to their WT littermates (2 months: genotype^*^time: *p* = 0.02, female-only: genotype^*^time: *p* = 0.02; 6 months: time: *p* < 0.0001, male-only –time: *p* = 0.02; female-only –time: *p* = 0.001; Supplementary Figure 3C, 4F). Interestingly, males of both genotypes did not improve at the task over time (Supplementary Figure 4E).

In late disease (12 months), BACHD mice performance is poorer than WT mice on the rotarod (genotype: *p* = 0.03). BACHD females perform worse than WT females (genotype: *p* = 0.0006). WT mice, but not BACHD mice, show a learning component at this age (time: *p* = 0.002). Overall, BACHD rotarod performance were not affected by weight. Approximately 7 to 20 mice of either combined or separated male/female groups are necessary to detect a 50 to 75% improvement in performance in early and mid-disease, respectively. Differences in late disease, using the accelerating rotarod, is detected in females only (12 females for a 75% treatment effect).

### Forelimb grip strength

Forelimb grip strength is indicative of fine motor skills which require neuromuscular function and muscle strength [61]. It is measured as the force required to break the mouse’s grip from a bar [45]. This method is quick, does not require animal training and provides information on muscle wasting and neuromuscular degeneration. However, body weight, stress, distraction, lack of motivation, the pulling angle and force applied by the experimenter can confound the grip strength measurement, so experimenter training is required to obtain consistent results [60, 62]. Historically, N171-82Q present decreased forelimb grip strength starting at 7 weeks old [63]. On the contrary, zQ175 have a normal forelimb grip strength throughout the disease while BACHD present an increase in grip strength at 2 months when their performance is normalized for their body weight [17, 64].

**Fig. 4:**
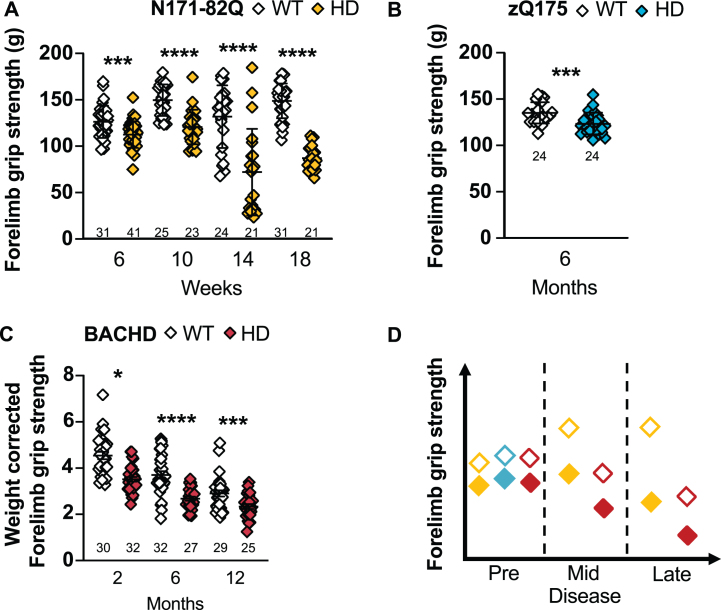
HD mice have a weaker forelimb grip strength. N171-82Q mice have a weaker forelimb grip strength throughout the disease (6 weeks: *t* = 3.693, *p* = 0.0002; 10 weeks: *t* = 5.619, *p* < 0.0001; 14 weeks: *t* = 4.973, *p* < 0.0001; 18 weeks: *t* = 12.94, *p* < 0.0001) (A) and zQ175 mice have a weaker forelimb grip strength early in the disease (6 months: *t* = 3.575, *p* = 0.0004) (B) compared to WT mice. C) After weight correction, BACHD have a decreased grip strength throughout the disease (2 months: *t* = 1.698, *p* = 0.05, 6 months: *t* = 5.711, *p* < 0.0001, 12 months: *t* = 3.229, *p* = 0.001). D) Representative model of the forelimb grips strength variation in the three mouse models as the disease progresses. All comparisons are made to WT mice of the same background. Sample sizes are presented under each group. HD, Huntington’s disease carrier; WT, wild-type. Data represent mean ± SEM. ^*^p ≤ 0.05, ^***^p ≤ 0.001, and ^****^p ≤ 0.0001 indicates a significant difference by a Student’s *t*-test for each time point.

N171-82Q mice present with a weaker forelimb grip strength throughout the disease (6 weeks: *p* = 0.0002, 10 to 18 weeks: *p* < 0.0001; Fig. 4A). Both N171-82Q males and females exhibit this weakness (males –6 weeks: *p* = 0.009, 10 weeks: *p* = 0.004, 14 weeks: *p* = 0.02, 18 weeks: *p* < 0.0001; females –6 weeks: *p* = 0.001; 10 to 18 weeks: *p* < 0.0001; Supplementary Figure 5A, B). Less than 6 to 18 N171-82Q mice of mixed-sex are required to detect a 50–75% improvement in mid- to late disease. Females require much smaller sample sizes (< 6–14) to detect the same difference while males require large groups.

Unlike earlier reports, we find that zQ175 mice forelimb grip strength is significantly reduced compared to WT in early disease (6 months: *p* = 0.0004; Fig. 4B), including when males and females are analyzed separately (male: *p* = 0.02, female: *p* = 0.007; Supplementary Figure 5C, D). These changes are detectable due to the small variance within groups, but weight is in part responsible for this difference. Also, large sample sizes are necessary to detect a 50% rescue in grip strength while about 20 mice are necessary to detect an 85–100% rescue in grip strength at this early stage.

In BACHD mice, the forelimb grip strength prior to weight correction is deceptive. Indeed, when forelimb grip strength is not corrected for the weight of the animals, BACHD are weaker in mid- (6 months: *p* = 0.03) and stronger in late disease (12 months: *p* = 0.007). However, when the forelimb grip strength is corrected for the animal weight, BACHD mice are significantly weaker throughout the disease (2 months: *p* = 0.05, 6 months: *p* < 0.0001, 12 months: *p* = 0.001; Fig. 4C). This holds true for males in early and mid-disease (2 months: *p* = 0.001; 6 months: *p* = 0.008; Supplementary Figure 5E) and at all timepoints in females (2 months: *p* < 0.0001; 6 months: *p* < 0.0001; 12 months: *p* = 0.001; Supplementary Figure 5F). This indicates a strong contribution of the increased weight to this measurement and grip strength in BACHD should always be weight corrected. Fourteen to 20 mice in mixed-sex groups and less than 6 to 14 females are recommended to detect the difference in grip strength in early and mid-disease.

### Descending rod

The descending rod or pole test consists of a vertical pole with a rough surface (Fig. 1B). The mouse is placed at the top and the time to start maneuvering down the pole (latency to descend), turning 180° to face downward (T-turn) and descend to the bottom of the pole (T-total) is recorded. This test was developed to measure the impact of lesions to the nigrostriatal pathway [65], affected in HD. This test requires mouse training and allows a rapid screen for disability and movement deficits. The R6/2 transgenic HD mouse model and Parkinson’s disease mouse models consistently take longer to descend the pole [66, 67]. However, there is no difference in the general climbing behavior in zQ175 throughout the disease while BACHD mice at 5 months take longer to start the descent [52, 68].

**Fig. 5:**
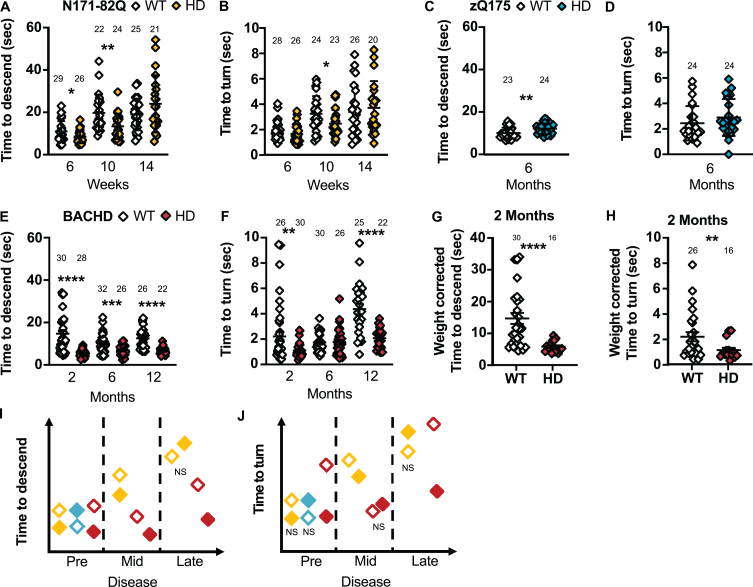
HD mice show variable performance on the descending rod. N171-82Q are faster to descend the rod in early- (*MWU* = 252, *p* = 0.02) and mid-disease (*t* = 2.873, *p* = 0.003) and to turn on the rod in mid-disease (*MWU* = 186, *p* = 0.03) (A, B). zQ175 mice take longer to descend the rod early in the disease (*t* = 2.828, *p* = 0.01) with no difference in the time to turn on the rod (C, D). BACHD mice are faster to descend on the rod throughout the disease (2 months: *MWU* = 95, *p* < 0.0001; 6 months: *t_*w*_* = 3.616, *p* = 0.0003; 12 months: *t_*w*_* = 5.987, *p* < 0.0001) and to turn on the rod in early (*MWU* = 220, *p* = 0.002) and late disease (*t_*w*_* = 5.155, *p* < 0.0001) (E, F). The difference in BACHD descending time (*MWU* = 65, *p* < 0.0001) and turn time on the rod (*MWU* = 174, *p* = 0.009) remains significant in early disease after correcting for weight (G, H). Representative model of the time taken to descend the rod or turn on the rod as the disease progresses (I, J). All comparisons are made to WT mice of the same background. Sample sizes are presented for each group. HD, Huntington’s disease carrier; NS, non-significant; WT, wild-type. Data represent mean ± SEM. ^*^p ≤ 0.05, ^**^p ≤ 0.01, ^***^p ≤ 0.001, and ^****^p ≤ 0.0001 indicates a significant difference by a Student’s *t*-test with or without Welch’s correction or Mann Whitney test for each time point.

Surprisingly, N171-82Q mice in early and mid-disease are quicker to turn downward and descend on the rod (T-turn –10 weeks: *p* = 0.03; T-total –6 weeks: *p* = 0.02, 10 weeks: *p* = 0.003; Fig. 5A). Further, N171-82Q mice in early and late disease are faster to start the descent (6 weeks: *MWU* = 109, *p* = 0.0006; 14 weeks: *MWU* = 127, *p* = 0.05). Similarly, males in early disease and females in mid-disease descend faster on the rod (Males –6 weeks: *p* = 0.003; Females: 10 weeks: *p* = 0.006; Supplementary Figure 6A, B) while males take longer to descend in late disease (14 weeks: *p* = 0.04). Male and female N171-82Q mice also start to descend earlier on the rod in early disease (6 weeks –males: *MWU* = 88.5, *p* = 0.02; females: *t_*w*_* = 4.526, *p* = 0.002) and in females, in late disease (*MWU* = 14, *p* = 0.004). Further, at 18 weeks, HD female mice could not be trained to perform the task altogether. The most sensitive measurement is the latency to descend in early disease which can be detected with less than 6 mice per genotype after a 50% normalization of the performance.

zQ175 take longer to descend on the rod (T-total: *p* = 0.01; Fig. 5C, D) and to start descending the rod (*MWU* = 146, *p* = 0.05) in early disease compared to WT mice. More specifically, males and females take longer to descend the rod (Male: *p* = 0.02; Female: *p* = 0.03; Supplementary Figure 6E, F) and males take longer to face downward on the rod (*p* = 0.01; Supplementary Figure 6G). The total time to descend is the most sensitive measurement and requires 20 to 25 mice to detect a complete recovery.

BACHD mice were markedly quicker to turn downward and descend the rod compared to WT mice in early and late disease (2 months –T-turn: *p* = 0.002, T-total: *p* < 0.0001; 6 months –T-total: *p* = 0.0003; 12 months –T-turn and T-total: *p* < 0.0001; Fig. 5E, F). Further, in early disease, the BACHD mice start the descent faster (*MWU* = 190, *p* = 0.002). Similarly, males and females are faster to descend throughout the disease (Male: 2 months: *p* = 0.0007; 6 months: *p* = 0.009; 12 months: *p* < 0.0001; Female –2 months: *p* < 0.0001, 6 months: *p* = 0.01, 12 months: *p* = 0.001; Supplementary Figure 6I, J). Further, males are faster to start the descent and turn on the rod in late disease (Latency: *MWU* = 11, *p* = 0.001; T-turn: *p* = 0.0002; Supplementary Figure 6K) while females are faster in early and late disease (Latency –2 months: *MWU* = 33.5, *p* = 0.004; T-turn –2 months: *p* = 0.004; 12 months: *p* = 0.008; Supplementary Figure 6L). Low sample sizes (less than 6 mice) are required to detect a 50% normalization of these behaviors in all groups in early and late disease.

Weight correction of these measurements in BACHD mice yielded similar results (2 months –Latency: *MWU* = 170.5, *p* = 0.005; T-turn: *p* = 0.009; T-total: *p* < 0.0001; Fig. 5G, H). However, BACHD mice tend to slide and fall off the rod more frequently than the other mouse models studied and take longer to learn the turning procedure which, without weight correction, can account for the high variance observed in these measurements.

### Narrow beam

The narrow beam, or raised beam, consists of crossing an elevated beam that progressively narrows to reach a safe platform (Fig. 1B). The time to cross, the number of paw slips and latency to start crossing are recorded. The beam is inclined upwards to promote spontaneous moving and reduce freezing on the beam [69]. This task reveals subtle motor deficits in motor coordination, gait and balance in a quantitative and sensitive manner that is partially redundant with the rotarod [19, 60]. Historically, N171-82Q mice in late disease (18 weeks) take longer to cross the narrow beam [63]. No change were detected in zQ175 performance at this task [58]. Surprisingly, although several studies report an increase in slip frequency in BACHD mice starting early (at 3 months) in the disease, none report a variance from WT mice in the time to cross [40, 70, 71]. Confounding factors of this task include reversal on the beam and pauses during crossing.

**Fig. 6:**
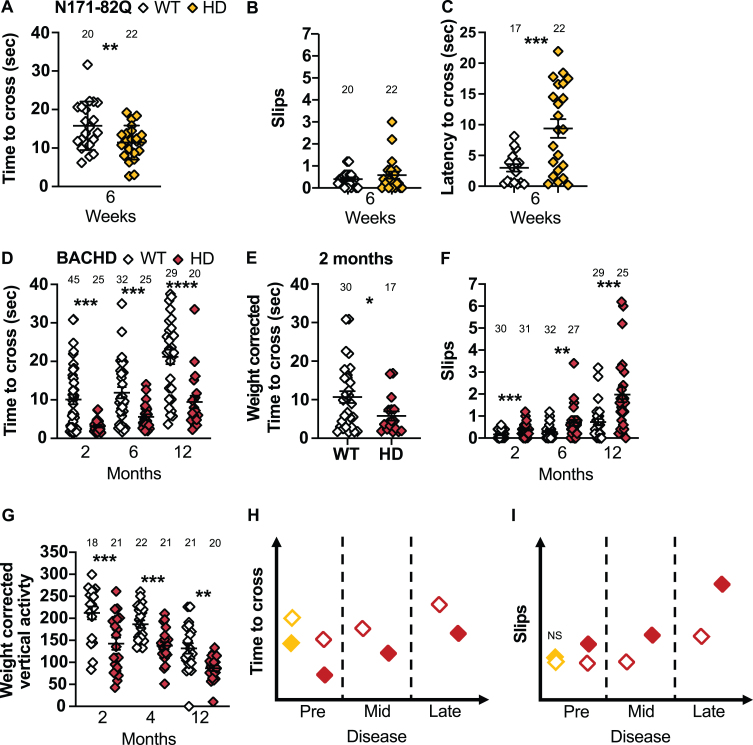
N171-82Q and BACHD mice perform differently on the narrow beam and climbing test. A) N171-82Q and BACHD mice cross the narrow beam faster early in the disease (*t* = 2.607, *p* = 0.006). The number of slips on the narrow beam do not differ in N171-82Q mice but they take longer to start to cross the beam (*t_*w*_* = 3.915, *p* = 0.0003) (B, C). D) BACHD mice cross the narrow beam faster throughout the disease (2 months: *MWU* = 446, *p* = 0.004, 6 months: *MWU* = 268, *p* = 0.006, 12 months: *t* = 4.440, *p* < 0.0001) (D) including after weight correction (2 months: *MWU* = 170, *p* = 0.03) (E). F) BACHD mice also slip more frequently while crossing the narrow beam (2 months: *MWU* = 241, *p* = 0.0003; 6 months: *MWU* = 239, *p* = 0.001; 12 months: *MWU* = 163, *p* = 0.0002). G) BACHD mice are also less active vertically (frequency of rearing and climbing) than WT mice during the climbing test (2 months: *t* = 3.465, *p* = 0.0007; 4 months: *t* = 4.077, *p* = 0.0001; 12 months: *t_*w*_* = 3.878, *p* = 0.001). Representative models of the time to cross and slips on the narrow beam as the disease progresses (H, I). All comparisons are made to WT mice of the same background. Sample sizes are presented for each group. HD, Huntington’s disease carrier; NS, non-significant; WT, wild-type. Data represent mean ± SEM. ^*^p ≤ 0.05, ^**^p ≤ 0.01, ^***^p ≤ 0.001, and ^****^p ≤ 0.001 indicates a significant difference by Student’s *t*-test with or without Welch’s correction or Mann Whitney test for each time point.

N171-82Q mice cross the narrow beam faster than WT mice in early disease (*p* = 0.006; Fig. 6A), especially females (*p* = 0.02; Supplementary Figure 7B). However, N171-82Q mice hesitate longer before crossing the narrow beam (*p* = 0.0003, Fig. 6C), including males (*p* = 0.0003; Supplementary Figure 7A). N171-82Q males also slip more frequently than WT mice while crossing (*p* = 0.01). The latency to cross is the most easily detectable measurement (25 mice for 50% normalization) in male groups.

BACHD mice, in early and mid-disease, cross the narrow beam faster than WT mice (2 months: *p* = 0.004, 6 months: *p* = 0.006, 12 months: *p* <0.0001; Fig. 6D) including after weight correction (2 months: *p* = 0.03; Fig. 6E). BACHD males also cross the beam faster throughout the disease (2 months: *p* = 0.03, 6 months: *p* = 0.0005, 12 months: *p* < 0.0001; Supplementary Figure 7C), while females do so in early and late disease only (2 months: *p* = 0.01; 12 months: *p* = 0.05; Supplementary Figure 7D). Further, BACHD mice of both sexes slip more frequently at all stages of the disease (2 months: *p* = 0.0003; 6 months: *p* = 0.001; 12 months: *p* = 0.0002; Fig. 6F). Males slip more frequently in mid-disease (6 months: *p* = 0.01) and females slip more frequently at all stages (2 months: *p* = 0.0002; 6 months: *p* = 0.02; 12 months: *p* = 0.0008). Finally, late-disease BACHD females start crossing the beam faster than WT (12 months: *t* = 1.965, *p* = 0.03). Although the change in crossing time is easy to detect (< 6–26 mice in mixed-sex groups or 7–11 males to detect a 50% rescue), this difference has never been reported previously. Until further testing has been conducted to explain this consistent faster crossing, it is preferable to measure number of slips, as this finding has been validated by multiple research groups.

### Climbing test

The climbing test consists of a wire mesh cylinder in which the mouse is placed (Fig. 1B). The frequency and duration of spontaneous vertical activity, rearing, standing on hind paws, and climbing was recorded [72]. Historically, R6/2 transgenic mice present a decrease in vertical activity starting at 4 weeks [59, 72] while BACHD mice take longer to start vertical activity at 4 weeks [59]. No change were reported in zQ175 performance at this task [17].

We find that BACHD mice present less frequent spontaneous vertical activity throughout the disease (2 months: *t* = 2.810, *p* = 0.004, 4 months *t* = 3.792, *p* = 0.0002, 12 months: *t* = 2.034, *p* = 0.002). These differences are amplified when the frequency and duration of the vertical activity is corrected for weight (Frequency: 2 months: *p* = 0.0007; 4 months: *p* = 0.0001; 12 months: *p* = 0.001; Duration: 2 months: *MWU* = 110, *p* = 0.01; 4 months: *t* = 1.655, *p* = 0.05; 12 months: *t* = 2.033, *p* = 0.02; Fig. 6G). While males present a decrease in weight-corrected frequency of vertical activity in early and mid-disease (2 months: *p* = 0.006; 4 months: *p* = 0.04; Supplementary Figure 7E), females show a decrease in frequency and duration of vertical activity corrected for weight throughout the disease (Frequency: 2 months: *p* = 0.006; 4 months: *p* = 0.0001; 12 months: *p* = 0.003; Duration: 2 months: *t* = 1.769, *p* = 0.05; 4 months: *t* = 1.749, *p* = 0.05; 12 months: *MWU* = 41, *p* = 0.02; Supplementary Figure 7F). The decrease in the frequency of vertical activity can be detected with 9–11 mice in mixed-sex groups for a full recovery at 2 months or a 75% recovery at 12 months. Smaller sample size are required to detect these changes in females. The climbing test measures should be corrected for weight for improved sensitivity.

### Activity chamber

The activity chamber uses photocell beams to measure motor ability through the distance travelled, frequency of jumping and the time spent rearing [69]. The performance in the activity chamber is evaluated as a total and in 10-min time bins to assess the activity progression over time. Exploration and stress influence the behavior in the activity chamber. Historically, N171-82Q mice are less active starting at 10 weeks while zQ175 males and BACHD mice are hypoactive at 20 weeks and 6 months respectively [17, 40, 73]. BACHD mice also jump less frequently starting at 2 months [64]. Finally, the rearing frequency is reduced in N171-82Q and BACHD mice starting at 16.5 weeks and 3 months respectively while remaining unchanged after a year in zQ175mice [52, 74, 75].

**Fig. 7:**
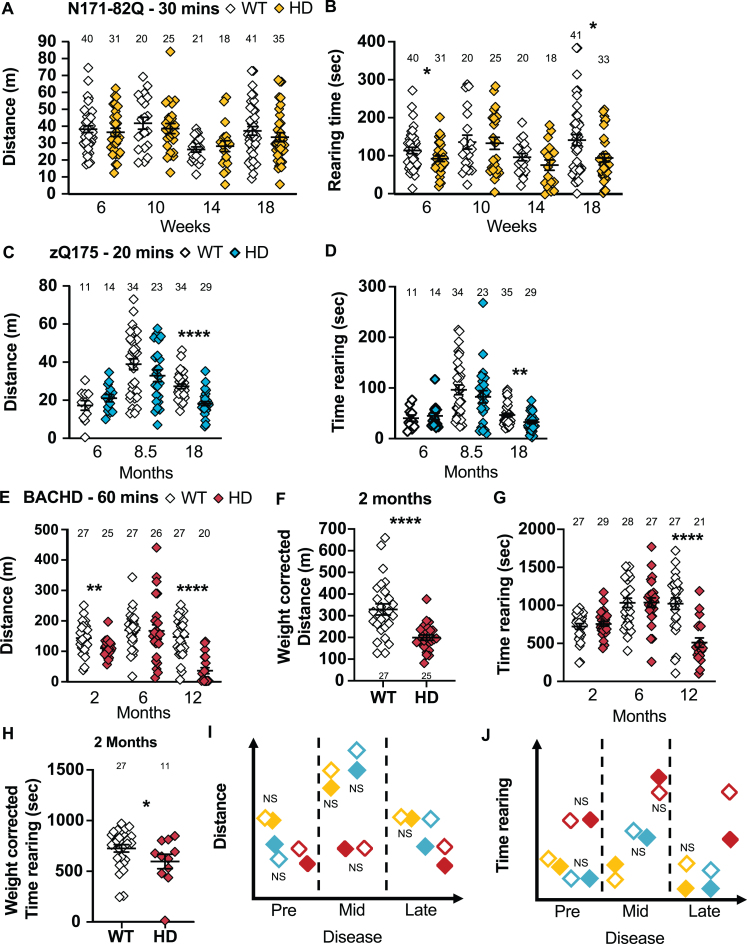
HD mice tend to travel a shorter distance and rear for a shorter time in the activity chamber. A) N171-82Q mice travel a similar distance in the activity chamber as WT mice but rear for a shorter time in early- (*t* = 1.894, *p* = 0.03) and late-disease (*MWU* = 500, *p* = 0.03) (A, B). zQ175 mice travel a shorter distance (*MWU* = 141, *p* < 0.0001) (C) and rear for shorter periods (*MWU* = 334, *p* = 0.01) (D) in the activity chamber in late disease. BACHD mice travel a shorter distance in early- (*t* = 2.938, *p* = 0.003) and late- (*MWU* = 46, *p* < 0.0001) disease in the activity chamber (E). F) In early disease, the decrease in distance travelled in BACHD is also detectable after weight correction (*t* = 4.583, *p* < 0.0001). G) BACHD mice also rear for shorter periods late in the disease only (*t* = 4.088, *p* < 0.0001). H) However, BACHD at two months show a decrease in the time spent rearing when correcting for weight (*t* = 1.760, *p* = 0.04). Representative models of the distance travelled and of the time spent rearing in the activity chamber as the disease progresses (I, J). All comparisons are made to WT mice of the same background. Sample sizes are presented for each group. HD, Huntington’s disease carrier; NS, non-significant; WT, wild-type. Data represent mean ± SEM. ^*^p ≤ 0.05, ^**^p ≤ 0.01, and ^****^p ≤ 0.0001 indicates a significant difference by a Student’s *t*-test or Mann-Whitney test for each time point.

There is no detectable difference in the distance traveled in the activity chamber over 30 min by N171-82Q mice throughout the disease (Fig. 7A), including when the distance travelled is separated into 10-min intervals. However, N171-82Q mice spend significantly less time rearing in early and late disease (6 and 18 weeks: *p* = 0.03; Fig. 7B) and jump less frequently in late-disease (18 weeks: *p* = 0.006; Supplementary Figure 8A) compared to WT mice. These differences are mainly influenced by males which travel a shorter distance in mid- to late disease (10 weeks: *p* = 0.02; 14 weeks: *p* = 0.03; Supplementary Figure 9A), spend less time rearing in mid- to late disease (10 weeks: *p* = 0.02; 14 weeks: *p* = 0.005; 18 weeks: *p* = 0.03; Supplementary Figure 9C) and jump less frequently after weight-correction in mid-disease (10 weeks: *p* = 0.03; Supplementary Figure 10A) compared to WT mice. N171-82Q females show a counterintuitive increase in distance travelled in mid-disease which then decreases in late disease (14 weeks: *p* = 0.003; 18 weeks: *p* = 0.05; Supplementary Figure 9B). HD females rear for longer in the mid-symptomatic stage (10 weeks: *p* = 0.006; Supplementary Figure 9D). Finally, HD females initially jump more frequently in early disease and less frequently by late disease (10 weeks: *p* = 0.03; 18 weeks: *p* = 0.002; Supplementary Figure 10B). Overall, females demonstrate a hyperactive phenotype in the activity chamber. These changes can be detected with large sample sizes due to large variance within groups.

In late disease, zQ175 mice travel shorter distances over 20 min in the activity chamber (*p* < 0.0001; Fig. 7C) including within 10-min time bins (genotype: *F_(1,62)_* = 25.74, *p* < 0.0001; time: *F_(1,62)_* =17.26, *p* < 0.0001; *post-hoc* WT vs. HD 0–10 min: *t* = 5.334, *p* < 0.0001; WT vs. HD 10–20 min: *t* = 3.554, *p* = 0.001). At this stage, zQ175 mice also spend less time rearing (*p* = 0.01; Fig. 7D, 8B). Similarly, males travel a shorter distance (*p* = 0.0002; Supplementary Figure 9E) including in 10-min time bins (18 months: genotype: *F_(1,30)_* = 16.20, *p* = 0.0004) and jump less frequently (*p* = 0.0006; Supplementary Figure 10C) at 18 months. Females travel shorter distances in late disease stages (18 months: *p* < 0.0008; 24 months: *p* = 0.0003; Supplementary Figure 9F), including in 10-min time bins (18 months: genotype: *F_(1,30)_* = 10.67, *p* < 0.003; time: *F_(1,30)_* = 51.12, *p* < 0.0001; *post-hoc* WT vs. HD 0–10 min: *t* = 6.880, *p* < 0.0001; WT vs. HD 10–20 min: *t* = 3.465, *p* = 0.003). At the same time, they rear for a shorter time overall (18 months: *p* = 0.02, 24 months: *p* < 0.0001; Supplementary Figure 9H) and jump less frequently (24 months: *p* < 0.0001; Supplementary Figure 10D). Taken together, the activity chamber discriminates late symptomatic zQ175 mice of both sexes using large mixed-sex groups (22 mice) while 11 to 13 males or females are required to detect a 50% amelioration of the phenotype.

BACHD mice travel a shorter distance over 60 min in the activity chamber in early and late disease (2 months: *p* = 0.003; 12 months: *p* < 0.0001; Fig. 7E), and these changes hold after weight correction (2 months: *p* < 0.0001; Fig. 7F). BACHD mice spend less time rearing in late disease (12 months: *p* < 0.0001; Fig. 7G), but even earlier after weight correction (2 months: *p* = 0.04, Fig. 7H). Finally, BACHD mice also jump less frequently at all the stages of the disease (Weight-corrected 2 and 6 months: *p* < 0.0001; 12 months: *p* < 0.0001; Supplementary Figure 8C, 8D). Males travel a shorter distance in the activity chamber in late disease (12 months: *p* = 0.0004; Supplementary Figure 9I). BACHD males also spend less time rearing in late disease (12 months: *p* = 0.03; Supplementary Figure 9J) and jump less frequently throughout the disease (Weight-corrected 2 months: *p* = 0.004; Weight-corrected 6 months: *p* = 0.04; 12 months: *p* = 0.0002; Supplementary Figure 10E, F). BACHD females travel a shorter distance in late disease (12 months: *p* = 0.0001; Supplementary Figure 9J), spend less time rearing in late disease (12 months: *p* = 0.0006; Supplementary Figure 9L) and jump less frequently throughout the course of disease (Weight-corrected 2 and 6 months: *p* < 0.0001; 12 months: *p* = 0.001; Supplementary Figure 10G, H). The same results were found if the first 30 min were analyzed separately, so a 30-min test is sufficient for this task. The activity chamber does not show major sex differences and the jumping frequency seems to be the most robust, predictive, and sensitive measurement within this assay. A 75% improvement can generally be detected by about 6–8 mice per group. At 2 and 6 months, the jumping frequency should however be corrected for weight.

## DISCUSSION

Three commonly used HD mouse models, the transgenic N171-82Q, the knock-in zQ175, and the full-length BACHD, were subjected to a comprehensive motor behavior test battery throughout their disease course. This battery included: weight tracking, rotarod performance, forelimb grip strength, descending rod, narrow beam crossing and activity chamber recording. Additionally, we assessed climbing activity in BACHD mice. Weight is an indicator of health in mouse models of human neurodegenerative disease and decreases with disease progression. Taken together, the motor tasks test strength, endurance, balance and fine motor, grasping capability, reflecting the neuromuscular function, coordination and gait requiring the corticostriatal and nigrostriatal pathways [19, 55, 61, 65].

To our knowledge, no formal inter-rater reliability has been measured on the specific tests measured here, but careful experimenter training and agreement on the specifics of the protocol are generally assumed. Also, we presume that a single experimenter will perform each task throughout the study. We recommend determining the test sequence starting with the most discriminatory task for a specific study mouse model and age. The rotarod test takes a few hours a day over four days to perform with a trial for mouse training on the first day of testing. The experimenter should stop the trial (record as latency to fall) when a mouse holds onto the rod without running for two consecutive rotations, as this behavior artificially inflates mouse performance. Motor task learning in the rotarod, as measured by the individual performance improvement during the four days of the assay, adds another layer of discrimination in this test. The forelimb grip strength and climbing test are measured within a few hours. Experimenters should practice the movement so that the mouse can grasp the rod and pull on it at the right angle (90°) without hitting the pole. The descending rod and narrow beam tests take a few hours over two days, one day for training and one for testing. The experimenter places the mouse upward on the descending rod and on the end of the narrow beam and gently prods them if they do not start the task after the pre-determined initial latency, generally 120 s. Finally, the activity chamber is the most hands off, with the mouse placed in the center of the activity chamber. Overall, the experimenter should note any external stressors and acclimate animals to rooms prior to testing. Handling mice for a few minutes over the course of a few days prior to the initial testing can minimize the handling stress. Notably, motivation can be an issue in behavior testing. Motivation is reduced in zQ175 and BACHD mice starting around 7 months and one year respectively [36]. When a mouse in early or mid-disease prematurely falls from the rotarod compared to similarly treated mice of the same group, presents a grip strength under 50 grams, or falls consistently from the descending rod or does not cross the narrow beam without being gently prodded several times, the trial should be excluded. In late disease, the phenotypes tend to be more variable, with the lack of motivation being the most difficult variable to dissect.

An increase in baseline stress, measured through circulating cortisol levels, correlates with patients’ total motor score and constitutes an early feature of HD [76]. This pathologic feature is replicated in R6/1 and R6/2 mouse models through an increase in the reaction to stress and a prolonged corticosterone responses [77, 78]. An increase in anxiety-like behavior was also reported in BACHD mice in early disease [59] and at approximately 8 months in zQ175 mice [79]. Whether the reduced descending rod descent time, narrow beam cross time, and initial hyperactivity detected in female N171-82Q are influenced by an altered stress response is unknown. Research to decouple the motor and anxiety-like behavior component of these tasks could help resolve these questions in the future.

The N171-82Q transgenic mice weigh less than their wildtype littermates and present a decrease in rotarod performance earlier than previously reported for this model (6 weeks instead of 10 weeks previously) [31]. Forelimb grip strength weakening was detected throughout the disease. On the descending rod, N171-82Q mice present a consistent decrease in the time to climb down the rod throughout the disease, contrary to what’s expected from the human pathology. On the narrow beam, these mice take longer to start crossing and slip more frequently as expected. However, they also cross the beam faster relative to WT mice, contrary to previous observations in late disease [63]. The most-commonly used transgenic model, R6/2 take longer to cross the beam starting early in the disease [15, 80] (Table 2). Potential co-factors which can exacerbate these counterintuitive phenotypes includes the stress response, hyperactivity or coordination deficits that prevent a proper performance on these tests [76, 78, 81]. Further, N171-82Q females could not be trained to perform the descending rod task at 18 weeks. Taken together, the descending rod is not optimal for phenotyping the N171-82Q mouse model while the number of paw slips and, in a lesser measure, the latency to start crossing are recommended to assess the performance on the narrow beam. Overall, the activity chamber task is most sensitive in N171-82Q males. All motor tests performed here detect the progressive motor phenotype at all stages of the disease, earlier than previously reported. Thus N171-82Q mice phenotypes are similar to those reported for R6/2 mice (Table 2). Overall, N171-82Q males are affected sooner and more consistently than females by the disease. This may justify the wide use of male N171-82Q mice in preclinical studies [32–35]. Data presented here can be used to update common practices in preclinical studies to target equal male and female representation.

**Table 2:** Comparison of previously reported motor deficits in HD mouse models

Model type	Transgenic fragment			Full-length knock-in		Transgenic full-length		
Mouse model	N171-82Q		Other model (R6/2)	zQ175		BACHD		Other model (YAC 128)
	Historically	This study		Historically	This study	Historically	This study
Weight	↓ 8 wks^14^	↓ 14 wks	↓ 9 wks^81^	↓ 12 mths^52^	↓ 6 mths	↑ 2 mths^25^	↑ 2 mths	↑ 8 mths^83^
	↓ 16 wks ♂^72^	↓ 10 wks ♂	↓ 6 wks ♂^84^	↓ 6.5 mths ♂^17^	↓ 6 mths ♂	↑ 2 mths ♂^25^	↑ 2 mths ♂	↑ 2 mths ♂^59^
	↓ 13 wks ♀^75^	↑ 10 wks ♀	↓ 14 wks ♀^84^	↓ 10 mths ♀^17^	↓ 6 mths ♀	↑ 2 mths ♀^25^	↑ 2 mths ♀	↑ 2 mths ♀^59^
		↓ 14 wks ♀					
Rotarod	Performance:	Performance:	Performance:	Performance:	Performance:	Performance:	Performance:	Performance:
	↓ 10 wks^31^	↓ 6 wks	↓ 8.5 wks^85^	textcolor [*rgb*] 0.00, 0.80, 0.10↓ 12 mth^59^	↓ 8.5 mths	↓ 1 mths^59^	↓ 2 mths	↓ 2 mths^86^
	↓ 16.5 wks ♂^72^	↓ 6 wks ♂			↓ 8.5 mths ♂	↓ 1 mth ♂^59^	↓ 2 mths ♂	↓ 2 mths ♂^59^
		↓ 10 wks ♀			↓ 24 mths ♀	↓1 mth ♀^59^	↓ 2 mths ♀	↓ 2 mths ♀^59^
	Learning:	Learning:	Learning:		Learning:		Learning:	Learning:
	↓ 12 wks ♂^14^	↓ 14 wks	↓ 14 wks^85^		=		↓ 2 mths	↓ 2 mths^86^
		↓ 14 wks ♂	↓ 14 wks ♂^85^		↓ 8.5 mths ♂		↓ 6 mths ♂
		↓ 14 wks ♀	↓ 14 wks ♀^85^		↑ 18 mths ♀		↓ 2 mths ♀
FGS	↓ 7 wks^63^	↓ 6 wks	↓ 7 wks^85^	=17	↓ 6 mths	↑ 2 mths –	↓ 2 mths	Not reported
		↓ 6 wks ♂			↓ 6 mths ♂	weight correct^64^	↓ 6 mths ♂
		↓ 6 wks ♀			↓ 6 mths ♀		↓ 2 mths ♀
							All weight correct
Descending rod	Not reported	↓ 6 wks	=	= Climbing^52^	↑ 6 mths	Latency descend:	↓ 2 mths	Climbing:
		↓ 6 wks ♂			↑ 6 mths ♂	↑ 5 mths^68^	↓ 2 mths ♂	↓ 3 mths^86^
		↓ 10 wks ♀			↑ 6 mths ♀		↓ 2 mths ♀	↓ 7 mths ♂^72^
Narrow beam	Cross time:	Cross time:	Cross time:	=58	NA	Slips:	Cross time:	Cross time:
	↑ 18 wks^63^	↓ 6 wks ♀	↑ 5 wks ♂^85^			↑ 3 mths^40^	↓ 2 mths	↑ 3 mths ♂^**72**^
	↑ 12 wks ♂^72^	Slips:					↓ 2 mths ♂
		↑ 6 wks ♂					↓ 2 mths ♀
		Latency:					Slips:
		↑ 6 wks					↑ 2 mths
		↑ 6 wks ♂					↑ 2 mths ♂
							↑ 6 mths ♀
Climbing test	NA	NA	Vertical act.	=17	NA	Vertical act.	Vertical act.	Vertical act.
			Frequency:			Latency:	Frequency:	Latency:
			↓ 4 wks^72^			↑ 4 mths^59^	↓ 2 mths	↑ 4 mths^59^
			Duration:				↓ 2 mths ♂
			↓ 4 wks^72^				↓ 2 mths ♀
			Latency:				Duration:
			↑ 4 wks^5972^				↓ 2 mths ♂
							↓ 2 mths ♀
Activity chamber	Distance:	Distance:	Distance:	Distance:	Distance:	Distance:	Distance:	Distance:
	↓ 10 wks ♂^73^	=	↓ 23 wks	↓ 5 mths ♂^17^	↓ 18 mths	↓ 6 mths ♂^40^	↓ 12 mths	↓ 3 mths^76^
		↓ 10 wks ♂	↓ 23 wks ♂^75^	=♀^17^	↓ 18 mths ♂		↓ 12 mths ♂	=♂^83^
		↑ 14 wks ♀	↓ 23 wks ♀^75^		↓ 18 mths ♀		↓ 12 mths ♀	↓ 12 mths ♀^83^
		↓ 18 wks ♀					
		Jumping:			Jumping:	Jumping:	Jumping:
		↓ 18 wks			=	↓ 2 mths^64^	↓ 2 mths
		↓ 10 wks ♂			↓ 8.5 mths ♂		↓ 2 mths ♂
		↑ 10 wks ♀			↓ 24 mths ♀		↓2 mths ♀
		↓ 18 wks ♀					Rearing:
	Rearing:	Rearing:	Rearing:	Rearing:	Rearing:	Rearing:	↓2 mths –	Rearing:
	↓ 16.5 wks^75^	↓ 18 wks	↓ 18 wks^85^	=12 wks^52^	↓ 18 mths	↓ 3 mths^74^	Weight correct	=♂^83^
		↓ 10 wks ♂	↓ 23 wks ♂^75^		=♂		↓ 2 mths ♂	=♀^83^
		↑ 10 wks ♀	↓ 23 wks ♀^75^		↓ 18 mths ♀		↓ 2 mths ♀

Green arrows represent expected behavior direction while red arrows indicate unexpected behavior direction. Mths, Months; act, activity; NA, Not available; wks, weeks.

The zQ175 knock-in model weigh less than their wildtype littermates consistently throughout the disease. This decrease in weight can also be detected earlier than formerly described (6 months vs. 12 months) [52]. We also detect a decrease in rotarod performance earlier than previously reported (8.5 months instead of 12 months previously) [58]. However, the late disease rotarod performance is dependent on the weight loss. zQ175 mice forelimb grip strength is weaker at 6 weeks, and influenced by the weight of the animals, while only a normal grip strength was detected previously [17]. Similarly, although some reports indicate normal climbing in zQ175 mice [52], we detect a subtle but significant decline in the descending rod performance starting in early disease. Finally, zQ175 mice hypoactivity, decrease in jumping and rearing is detectable in late disease, a phenotype not previously described to our knowledge [17]. Overall, zQ175 mice present subtle but detectable motor impairments in early disease due to the small variance between individuals. Consequently, single-sex groups are recommended as they require smaller sample sizes to detect motor differences than mixed-sex groups. The decline in motor abilities is detectable though a distinct range of discriminatory tests throughout the disease (Table 3). As zQ175 is the only knock-in model presenting a consistent hemizygous pathology, we do not compare this model’s performance to other knock-in models (Table 2).

**Table 3:** Motor changes over disease progression in three HD mouse models

Mouse model	N171-82Q				zQ175			BACHD		
Disease stage	Early	Mid	Late	Late	Early	Mid	Late	Early	Mid	Late
Age (Unit)	6	10	14	18	6	8.5	18	2	6	12
	Weeks				Months			Months		
Weight	=	=	↓↓↓↓	↓↓↓↓	↓	↓↓	↓↓↓↓	↑↑ ↑↑	↑↑ ↑↑	↑↑ ↑↑
Rotarod Performance	↓↓	↓↓	↓↓	↓↓↓↓	=	↓	↓	↓↓↓↓	↓↓↓↓	↓↓↓
Rotarod Learning	=	=	↓↓↓↓	=	=	=	=	↓↓	↓↓↓↓	↓↓
FGS	↓↓↓	↓↓↓↓	↓↓↓↓	↓↓↓↓	↓↓↓	NA	NA	↓*	↓↓↓↓*	↓↓↓*
DR – T-turn	=	↓	=	NC	=	NA	NA	↓↓	=	↓↓ ↓↓
DR – T-total	↓	↓↓	=	NC	↑↑	NA	NA	↓↓ ↓↓	↓↓ ↓	↓↓ ↓↓
DR – Latency	↓↓ ↓	=	↓	NC	↑	NA	NA	↓↓	=	=
NB – Cross time	↓↓	NA	NA	NA	NA	NA	NA	↓↓ ↓	↓↓ ↓	↓↓ ↓↓
NB – Slips	=	NA	NA	NA	NA	NA	NA	↑↑↑	↑↑	↑↑↑
NB – Latency	↑↑↑	NA	NA	NA	NA	NA	NA	=	=	=
CT – Frequency	NA	NA	NA	NA	NA	NA	NA	↓↓↓	↓↓↓	↓↓
AC – Distance	=	=	=	=	=	=	↓↓↓↓	↓↓	=	↓↓↓↓
AC – Jump	=	=	=	↓↓	=	=	=	↓↓↓↓*	↓↓↓↓*	↓↓↓↓
AC – Rearing	↓	=	=	↓	=	=	↓↓	=	=	↓↓↓↓
Recommended	Rotarod	Rotarod	Weight	Weight	Weight	Weight	Weight	*Weight*	*Weight*	*Weight*
	FGS	FGS	Rotarod	Rotarod	FGS	Rotarod	Rotarod	Rotarod	Rotarod	Rotarod
	*DR*	*DR*	FGS	FGS	DR		AC	FGS	FGS	FGS
	NB		*DR*	AC				*DR*	*DR*	*DR*
	AC							NB	NB	NB
								CT	CT	CT
								AC	AC	AC

Black arrows represent expected behavior direction while red arrows indicate behavior that are not following the human HD pathology. Italicized recommended tests indicate unexpected result direction. *Weight-corrected results; AC, Activity chamber; CT, Climbing test; DR, Descending rod; FGS, Forelimb grip strength; NA, Not available; NB, Narrow beam; NC, Mice were not capable to perform the task; T-time, Time to descend the descending rod; T-turn, Time to turn downward on the descending rod.

The full-length mouse models fail to replicate the weight loss observed in HD patients. These models are obese, starting at an early age (2 months) in BACHD and later, starting at 8 months, in the other available full-length model, YAC128 [82, 83] (Table 2). Consequently, some full-length HD models’ motor performances are influenced by body weight and a weight correction is recommended when assessing forelimb grip strength and jumping behavior up to 6 months. BACHD mice present a decrease in rotarod performance in early disease. Contrary to HD patients, BACHD mice historically present an increase in forelimb grip strength at 2 months [64]. However, when grip strength is corrected for the body weight, these animals are effectively weaker throughout the disease. Regarding the descending rod, historical data indicate that BACHD mice take longer to start the descent at 5 months old [68]. In our hands however, BACHD present a consistent decrease in the time to climb down the rod throughout the disease. Further, BACHD mice also consistently cross the narrow beam faster than their WT counterparts. Interestingly, prior reports of BACHD mice performance on the narrow beam do not report the time to cross [40, 70, 71]. BACHD mice also consistently slipped more frequently on the narrow beam, in accordance to previously published work [40]. BACHD mice present a high within-group variability in both these tests. Automated measurements on the narrow beam could help parse out detailed performances on the varying narrow beam widths. The climbing test constitutes an appealing alternative to the descending rod and narrow beam as the decrease in vertical activity is robust throughout the disease, especially after correcting for weight. Regarding the activity chamber, BACHD mice are generally hypoactive, jump less frequently and rear less in early disease. With the new information presented in this manuscript, YAC128 mice present a slightly slower disease progression compared to BACHD mice (Table 3). The BACHD mice decrease in motor abilities is easily detectable in most tasks presented here and throughout the disease progression. The only exception is the descending rod that will require further testing to be fully understood.

The test battery presented here is sensitive and requires minimal time and training. In addition to this, we also issue recommendations on the most discriminatory assays at the different disease stages in three representative HD mouse models and the associated sample sizes required to detect different extents of therapeutic benefits (Tables 1 and 3). We recommend the use of both sexes to detect sex-specific therapeutic treatment effects, and we find that analyzing the sexes separately reduces variance within the groups and therefore the required sample size (Supplementary Tables 1-4). In summary, our data present a guide for designing preclinical studies that include a motor component in HD mouse models.

## Supplementary Material

Supplementary Material
