# Supplementary material for: Temporal Phenotypic Changes in Huntington’s Disease Models for Preclinical Studies
Source: J Huntingtons Dis. 2022 Mar 1;11(1):35–57. doi: 10.3233/JHD-210515 (PMC9028736; doi:10.3233/JHD-210515)
Supplement: Supplementary Material [file jhd-11-jhd210515-s001.pdf]

Supplementary Material

Temporal Phenotypic Changes in Huntington's Disease Models for Preclinical Studies

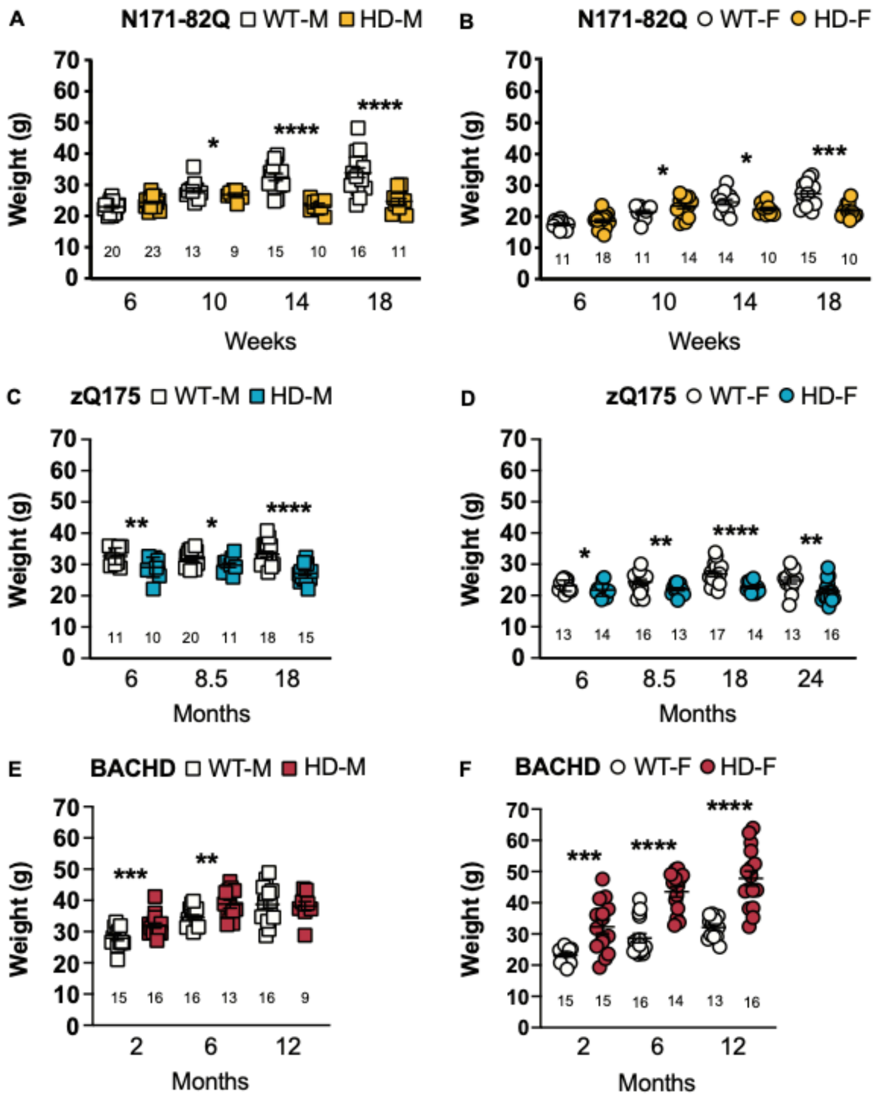

Supplementary Figure 1. Male and female HD mice show differences in weight throughout the disease. A) N171-82Q males are lighter in mid- to late disease (10 weeks: $MWU = 32.5$, $p = 0.04$; 14 weeks: $t_w = 7.189$, $p < 0.0001$; 18 weeks: $t_w = 4.998$, $p < 0.0001$). B) N171-82Q females are initially heavier in mid-disease (10 weeks: $MWU = 42.5$, $p = 0.03$) and become lighter in late disease (14 weeks: $t = 2.002$, $p = 0.03$; 18 weeks: $t = 4.190$, $p = 0.0002$). zQ175 males and females are lighter throughout the disease (Male - 6 months: $t = 2.781$, $p = 0.006$, 8.5 months: $t = 2.232$, $p = 0.02$, 18 months: $t = 5.536$, $p < 0.0001$; Females - 6 months: $t = 1.875$, $p = 0.04$, 8.5 months: $t_w = 2.666$, $p = 0.007$, 18 months: $t_w = 4.523$, $p < 0.0001$, 24 months: $t = 2.676$, $p = 0.006$) (C, D). BACHD males are heavier in early ($MWU = 42$, $p = 0.0007$) and mid-disease ($t = 3.020$, $p = 0.003$) while BACHD females are heavier throughout the disease (2 months: $t_w = 4.190$, $p = 0.0003$; 6 months: $MWU = 14$, $p < 0.0001$; 12 months: $t_w = 6.125$, $p < 0.0001$) (E, F). All comparisons are made to WT mice of the same background. Data represent mean ± SEM. Sample sizes are presented under each group. HD, Huntington's disease carrier; F, female; M, male; WT, wild-type. $*p \leq 0.05$, $**p \leq 0.01$, $***p \leq 0.001$, and $****p \leq 0.0001$ indicate a significant difference by a Student's t-test with or without Welch's correction or a Mann-Whitney test for each sex and time point.

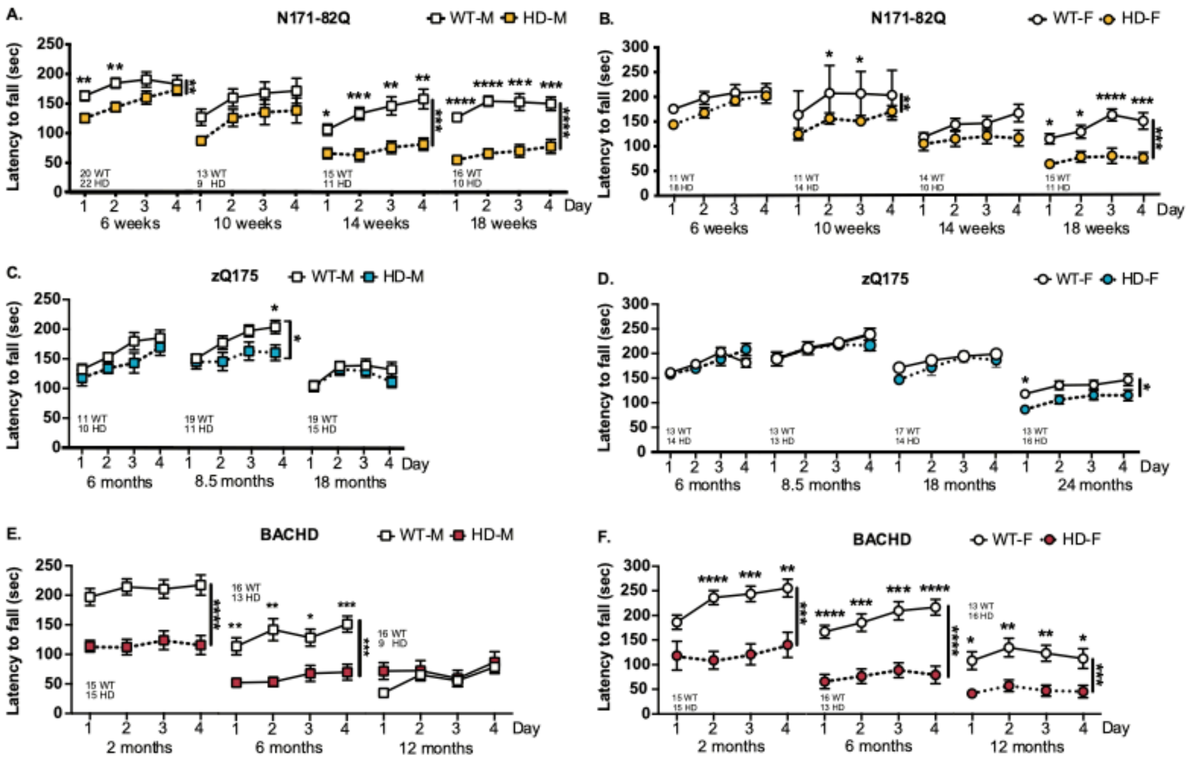

Supplementary Figure 2. HD male and female mice separately generally perform poorly on the accelerating rotarod. A) N171-82Q male mice perform poorly on the rotarod in early (Genotype: $F_{(1,40)} = 7.581$, $p = 0.009$; Time: $F_{(2,74)} = 7.195$, $p = 0.002$, Post-hoc Day 1-2: $t = 3.371$, $p = 0.007$) and late-disease (14 weeks: Genotype: $F_{(1, 24)} = 15.18$, $p = 0.0007$, Time: $F_{(2, 54)} = 11.96$, $p < 0.0001$, Post-hoc Day 1-4: $t = 3.027$, $p = 0.02$; 18 weeks: Genotype: $F_{(1,24)} = 33.84$, $p < 0.0001$; Time: $F_{(2,59)} = 5.682$, $p = 0.003$, Post-hoc Day 1-4: $t = 4.357$, $p = 0.001$). B) N171-82Q fall earlier from the rotarod in mid- (Genotype: $F_{(1, 23)} = 9.098$, $p = 0.006$, Time: $F_{(3,69)} = 10.27$, $p < 0.0001$, Post-hoc Day 2-3: $t = 2.820$, $p = 0.05$) and late disease (Genotype: $F_{(1,24)} = 14.07$, $p = 0.001$, Time: $F_{(3,72)} = 11.99$, $p < 0.0001$, Post-hoc Day 1-4: $t = 2.726$, $p = 0.05$). zQ175 male mice perform poorly in mid-disease (Genotype*Time: $F_{(3,84)} = 3.144$, $p = 0.03$; Post-hoc Day 4: $t = 2.544$, $p = 0.05$) (C) while zQ175 females perform poorly in late disease compared to WT females (24 months - Genotype: $F_{(1,27)} = 6.283$, $p = 0.02$, Time: $F_{(3,69)} = 11.35$, $p < 0.0001$, Post-hoc Day 1: $t = 3.251$, $p = 0.01$) (D). BACHD males perform poorly on the rotarod in early (Genotype: $F_{(1,28)} = 27.77$, $p < 0.0001$) and mid-disease (Genotype: $F_{(1,27)} = 18.67$, $p = 0.0002$; Time: $F_{(3,75)} = 3.794$, $p = 0.02$, Post-hoc Day 1-4: $t = 3.068$, $p = 0.02$) (E) while BACHD females fall earlier from the rotarod throughout the disease (2 months - Genotype: $F_{(1,28)} = 17.71$, $p = 0.0002$, Time: $F_{(2,57)} = 7.044$, $p = 0.002$, Post-hoc Day 2-4: $t = 3.694$, $p = 0.004$; 6 months - Genotype: $F_{(1,28)} = 32.63$, $p < 0.0001$, Time: $F_{(2,69)} = 6.820$, $p = 0.001$, Post-hoc Day 1-4: $t = 4.716$, $p = 0.0002$; 12 months - Genotype: $F_{(1,27)} = 14.94$, $p = 0.0006$, Time: $F_{(3,73)} = 3.273$, $p = 0.03$; Post-hoc Day 1-4: $t = 2.819$, $p = 0.04$) (F). All comparisons are made to WT mice of the same background. Data represent mean ± SEM. Sample sizes are presented under each group. HD, Huntington's disease carrier; F, female; M, male; WT, wild-type. *$p \leq 0.05$, **$p \leq 0.01$, ***$p \leq 0.001$, and ****$p \leq 0.0001$ indicate a significant difference by a 2-way repeated measure ANOVA with Sidak's multiple comparison tests for each time point. Side bars indicate a genotype effect. Side brackets indicate a genotype and time interaction.

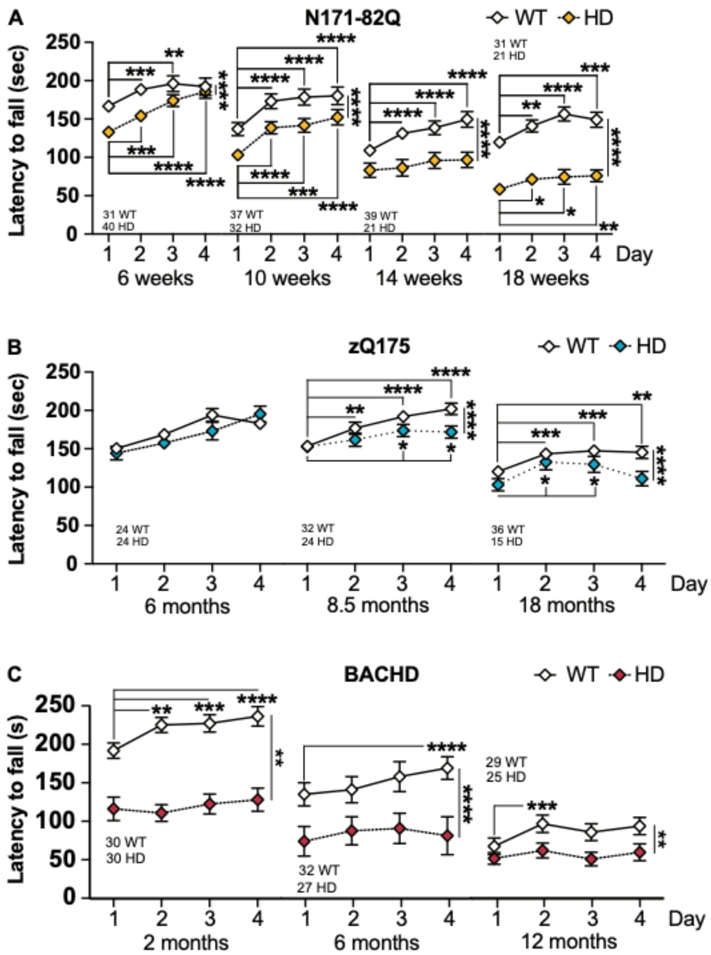

Supplementary Figure 3. Two HD mouse models fail to improve their performance on the accelerating rotarod. A) N171-82Q mice fail to improve on the motor task in late-disease compared to WT mice (14 weeks – Genotype*Time: $F_{(3,72)} = 3.678$, $p = 0.02$, Post-hoc WT Day 1-4: $t = 5.620$, $p < 0.0001$). zQ175 mice show no difference in learning of the motor task (B) while BACHD mice do not improve with repetition of the motor task throughout the disease compared to WT mice (2 months – Genotype*Time: $F_{(3,174)} = 3.222$, $p = 0.02$, Post-hoc WT Day 1-4: $q = 5.186$, $p < 0.0001$; 6 months - Genotype: $F_{(1,57)} = 43.26$, $p < 0.0001$, Time: $F_{(3,171)} = 9.597$, $p < 0.0001$, Post-hoc WT Day 1-4: $q = 7.730$, $df = 171$, $p < 0.0001$, $d = 0.7$; 12 months - Genotype: $F_{(1,52)} = 5.055$, $p = 0.03$, Time: $F_{(2,130)} = 5.680$, $p = 0.002$, Post-hoc WT Day 1-2: $q = 4.674$, $df = 28$, $p = 0.0002$, $d = 0.5$) (C). All comparisons are made to WT mice of the same background. Data represent mean ± SEM. Sample sizes are presented under each group. WT, wild-type; HD, Huntington's disease carrier. *$p \leq 0.05$, **$p \leq 0.01$, ***$p \leq 0.001$, and ****$p \leq 0.0001$ indicate a significant difference by a 2-way repeated measure ANOVA with Dunnett's multiple comparison tests for each time point. Side bars indicate a time effect.

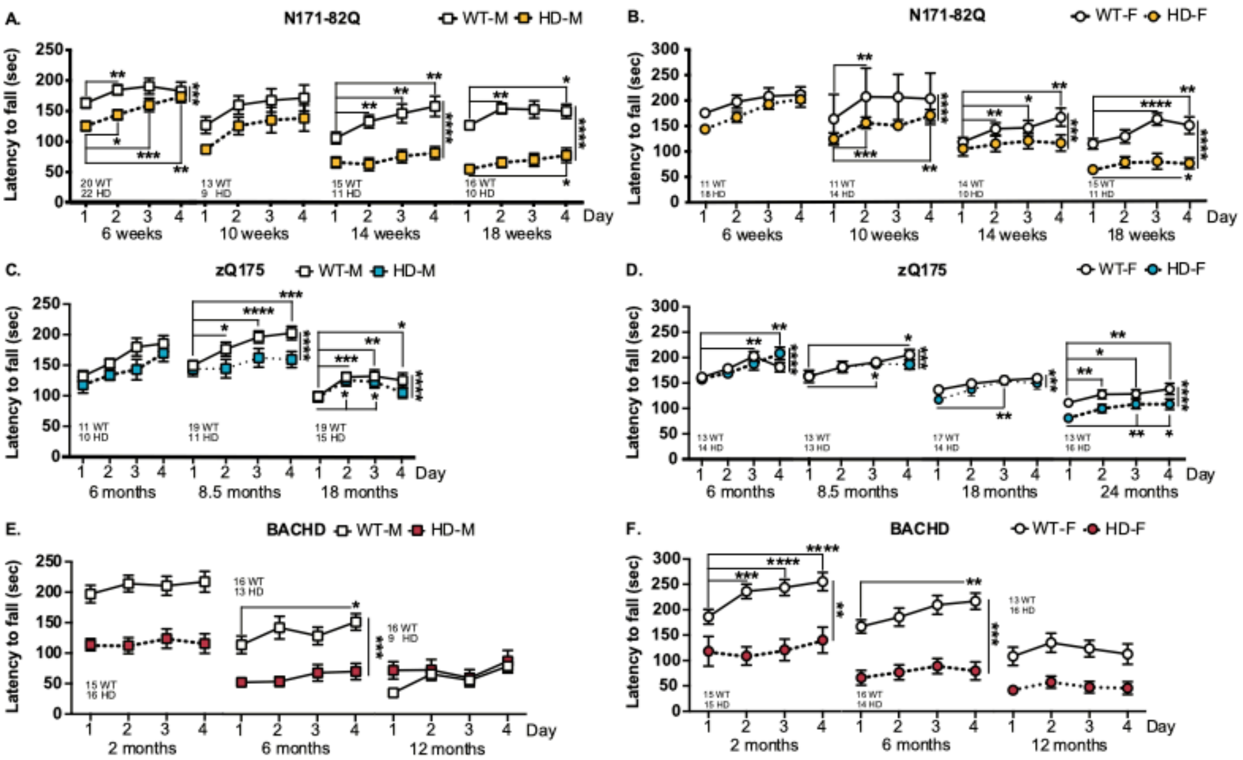

Supplementary Figure 4. HD male and female mice separately generally fail to improve their performance on the accelerating rotarod. N171-82Q males and females fail to improve at the motor task in mid-disease when compared to WT mice (Male: 14 weeks – Genotype*Time: $F_{(3,72)} = 3.678$, $p = 0.02$, Post-hoc WT Day 1-4: $q = 4.203$, $p = 0.002$; Female: 14 weeks – Genotype*Time: $F_{(3,66)} = 3.071$, $p = 0.03$, Post-hoc WT Day 1-4: $q = 4.321$, $p = 0.002$) (A, B). zQ175 male fail to improve their performance on the rotarod in mid-disease when compared to WT mice (8.5 months – Time: $F_{(3,84)} = 12.88$, $p < 0.0001$, Post-hoc WT Day 1-4: $q = 3.198$, $p = 0.01$) (C) while zQ175 females improve their performance at the motor task over time in late disease while WT females do not (18 months – Time: $F_{(3,77)} = 6.915$, $p = 0.0006$, Post-hoc zQ175 Day 1-3: $q = 3.650$, $p = 0.008$) (D). BACHD male mice present decreased motor improvement after repetition at a mid-disease time point (Genotype: $F_{(1,27)} = 18.67$, $p = 0.0002$, Time: $F_{(3,75)} = 3.794$, $p = 0.02$, Post-hoc WT Day 1-4: $q = 4.289$, $df = 15$, $p = 0.04$, $d = 0.7$) (E) while BACHD females fail to improve their performance on the accelerated rotarod in early- (Genotype*Time: $F_{(3,84)} = 3.630$, $p = 0.02$, Post-hoc WT Day 1-4: $q = 6.506$, $p < 0.0001$) and mid-disease (Genotype: $F_{(1,28)} = 32.63$, $p < 0.0001$, Time: $F_{(2,69)} = 6.820$, $p = 0.001$, Post-hoc WT Day 1-4: $q = 5.258$, $p = 0.01$) (F). All comparisons are made to WT mice of the same background. Data represent mean ± SEM. Sample sizes are presented under each group. HD, Huntington's disease carrier; F, female; M, male; WT, wild-type. $^{*}p \leq 0.05$, $^{**}p \leq 0.01$, $^{***}p \leq 0.001$, and $^{****}p \leq 0.0001$ indicate a significant difference by a 2-way repeated measure ANOVA with Dunnett's or Tukey's multiple comparison tests for each time point. Side bars indicate a time effect.

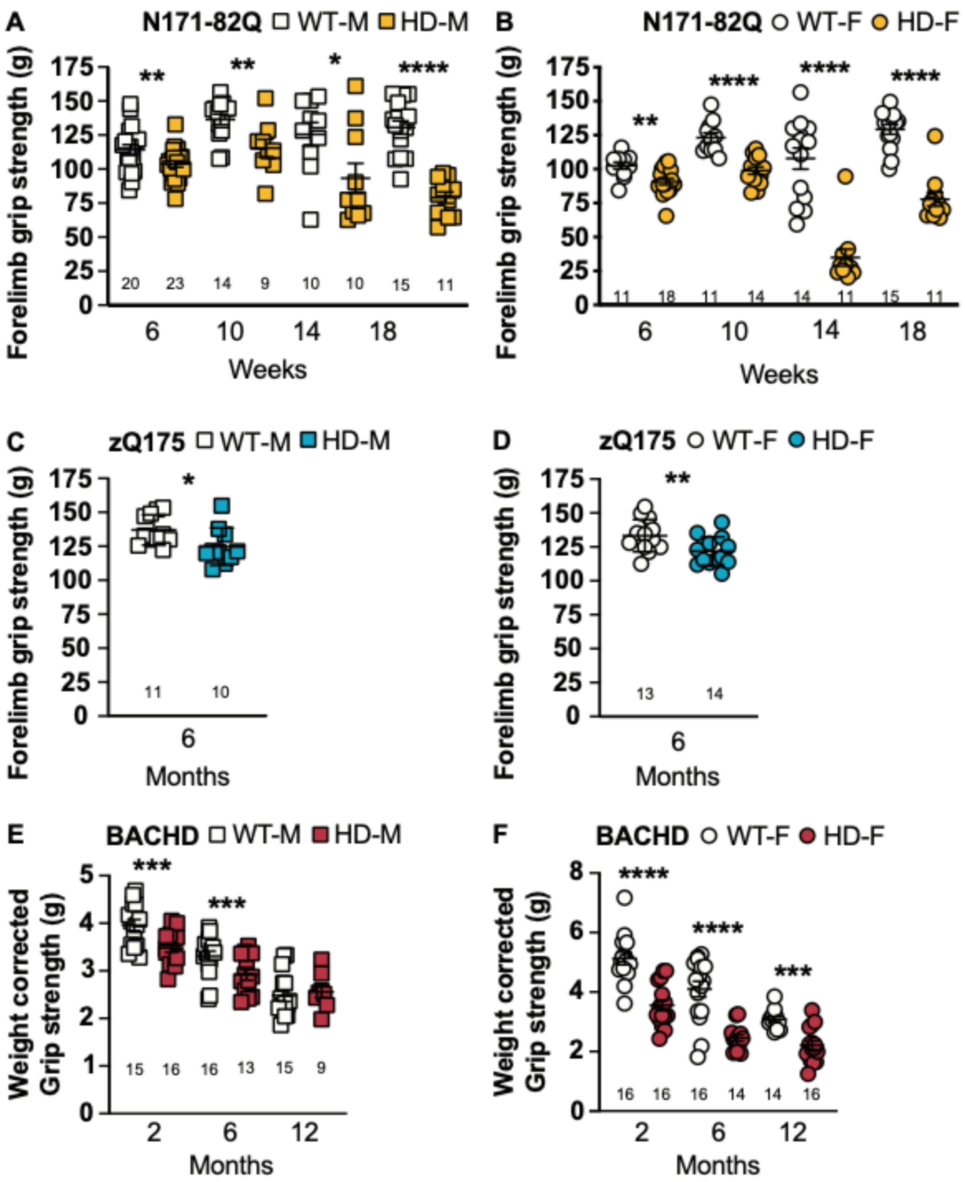

Supplementary Figure 5. HD mouse models of both sexes have a weaker forelimb grip strength. N171-82Q male and female mice have a weaker forelimb grip strength throughout the disease (Male – 6 weeks: $t = 2.464$, $p = 0.009$; 10 weeks: $t = 2.995$, $p = 0.004$; 14 weeks: $t = 2.294$, $p = 0.02$; 18 weeks: $t = 7.679$, $p < 0.0001$; Females - 6 weeks: $t = 3.315$, $p = 0.001$; 10 weeks: $t = 5.546$, $p < 0.0001$; 14 weeks: $MWU = 5$, $p < 0.0001$; 18 weeks: $MWU = 4$, $p < 0.0001$) (A, B). zQ175 males and females have a decreased grip strength early in the disease (Male: $t = 2.26$, $p = 0.02$; Female: $t = 2.664$, $p = 0.007$) (C, D). BACHD males are weaker in early and mid-disease (2 months: $t = 3.307$, $p = 0.001$; 6 months: $t = 2.591$, $p = 0.008$) while females are weaker throughout the disease (2 months: $t = 5.770$, $p < 0.0001$; 6 months: $t = 5.750$, $p < 0.0001$; 12 months: $t = 5.181$, $p = 0.001$) when their forelimb grip strength is corrected for weight (E, F). All comparisons are made to WT mice of the same background. Data represent mean ± SEM. Sample sizes are presented under each group. HD, Huntington's disease carrier; F, female; M, male; WT, wild-type. $^{*}p \leq 0.05$, $^{**}p \leq 0.01$, $^{***}p \leq 0.001$, and $^{****}p \leq 0.0001$ indicate a significant difference by a Student's t-test with or without Welch's correction or Mann Whitney test for each time point.

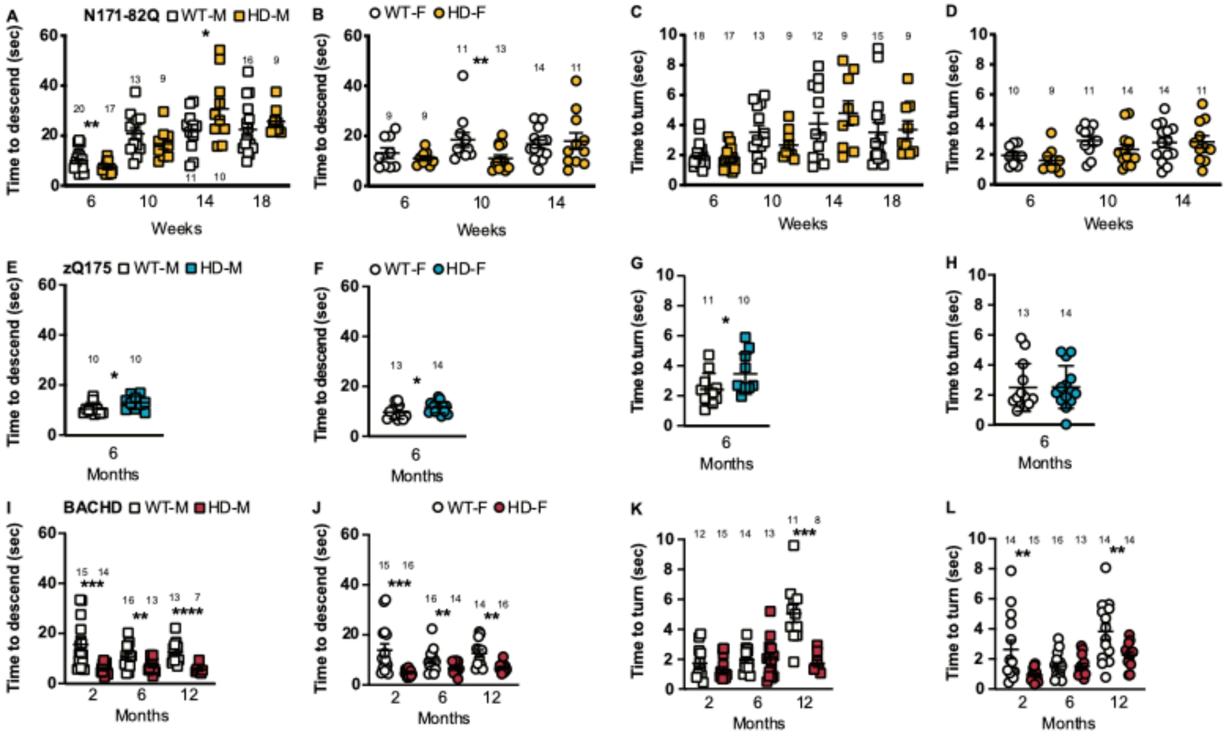

Supplementary Figure 6. HD mouse models of both sexes show differences in their descending rod performance. A) N171-82 males are faster to descend the rod in early disease (t_w = 2.925, p = 0.003), and this time lengthens at a late disease stage (14 weeks: t = 1.835, p = 0.04) compared to WT males. B) N171-82Q females are faster to descend the rod in mid-disease (*MWU* = 29, p = 0.006). N171-82Q males and females show a similar time to turn on the rod throughout the disease compared to WT mice (C, D). zQ175 males and females are slower to descend the rod in early disease (Male: *MWU* = 23, p = 0.02; Female: t = 1.939, p = 0.03) (E, F). zQ175 males are slower to turn on the rod in early disease (t = 1.969, p = 0.01) (G) while females show no such difference (H). BACHD males and females are faster to descend the rod throughout the disease (Male – 2 months: t_w = 3.924, p = 0.0007; 6 months: t = 2.508, p = 0.009; 12 months: *MWU* = 3, p < 0.0001; Female – 2 months: *MWU* = 25, p < 0.0001; 6 months: t_w = 2.427, p = 0.01; 12 months: t_w = 3.650, p = 0.001) (I, J). K) BACHD males in late disease take longer to turn downward on the rod (t_w = 5.108, p = 0.0002). L) BACHD females take less time to turn on the rod in early (*MWU* = 46, p = 0.004) and late-disease (t_w = 2.698, p = 0.008). All comparisons are made to WT mice of the same background. Sample sizes are presented for each group. HD, Huntington's disease carrier; F, female; M, male; WT, wild-type. Data represent mean ± SEM. *$p \leq 0.05$, **$p \leq 0.01$, ***$p \leq 0.001$, and ****$p \leq 0.0001$ indicate a significant difference by a Student's t-test with or without Welch's correction or Mann Whitney test for each time point.

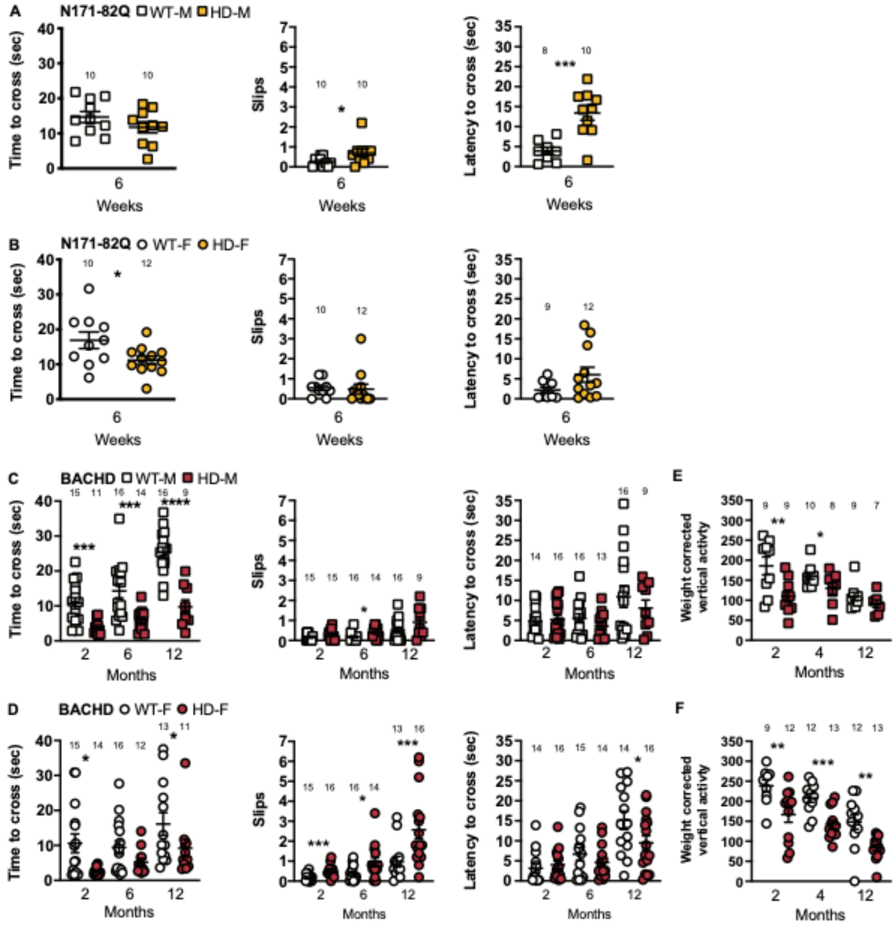

Supplementary Figure 7. HD mice of both sexes show differences in their performance on the narrow beam and climbing test. A) Early in the disease, N171-82Q males take a similar amount of time to cross the narrow beam but slip more frequently ($MWU = 21$, $p = 0.01$) and take longer to start crossing ($t = 4.297$, $p = 0.0003$) the narrow beam. B) N171-82Q females in early disease are faster to cross ($t = 2.297$, $p = 0.02$,) but present no difference in the number of slips or latency to start crossing the narrow beam. C) BACHD males take less time to cross the narrow beam throughout the disease (2 months: $MWU = 65$, $p = 0.03$; 6 months: $t_w = 3.818$, $p = 0.0005$; 12 months: $t = 6.057$, $p < 0.0001$) and slip more frequently in mid-disease ($MWU = 53$, $p = 0.01$) compared to WT males. D) BACHD females are faster to cross the narrow beam in early ($MWU = 55$, $p = 0.01$) and late-disease ($MWU = 42$, $p = 0.05$,), slip more frequently throughout the disease (2 months: $MWU = 37$, $p = 0.0002$; 6 months: $MWU = 61$, $p = 0.02$; 12 months: $MWU = 35$, $p = 0.0008$) and start to cross the narrow beam faster in late disease ($t = 1.965$, $p = 0.03$). BACHD males are less active vertically than WT males during the climbing test in early ($t = 2.849$, $p = 0.006$) and mid-disease ($t = 1.921$, $p = 0.04$) (E) while BACHD females are less active vertically when compared to WT females throughout the disease (2 months: $t = 2.771$, $p = 0.006$; 4 months: $t = 4.400$, $p = 0.0001$; 12 months: $t = 3.241$, $p = 0.003$) after correcting for weight (F). All comparisons are made to WT mice of the same background. Sample sizes are presented under each group. HD, Huntington's disease carrier; F, female; M, male; WT, wild-type. Data represent mean ± SEM. *$p \leq 0.05$, **$p \leq 0.01$, ***$p \leq 0.001$, and ****$p \leq 0.0001$ indicate a significant difference by a Student's t-test with or without Welch's correction or Mann Whitney test for each time point.

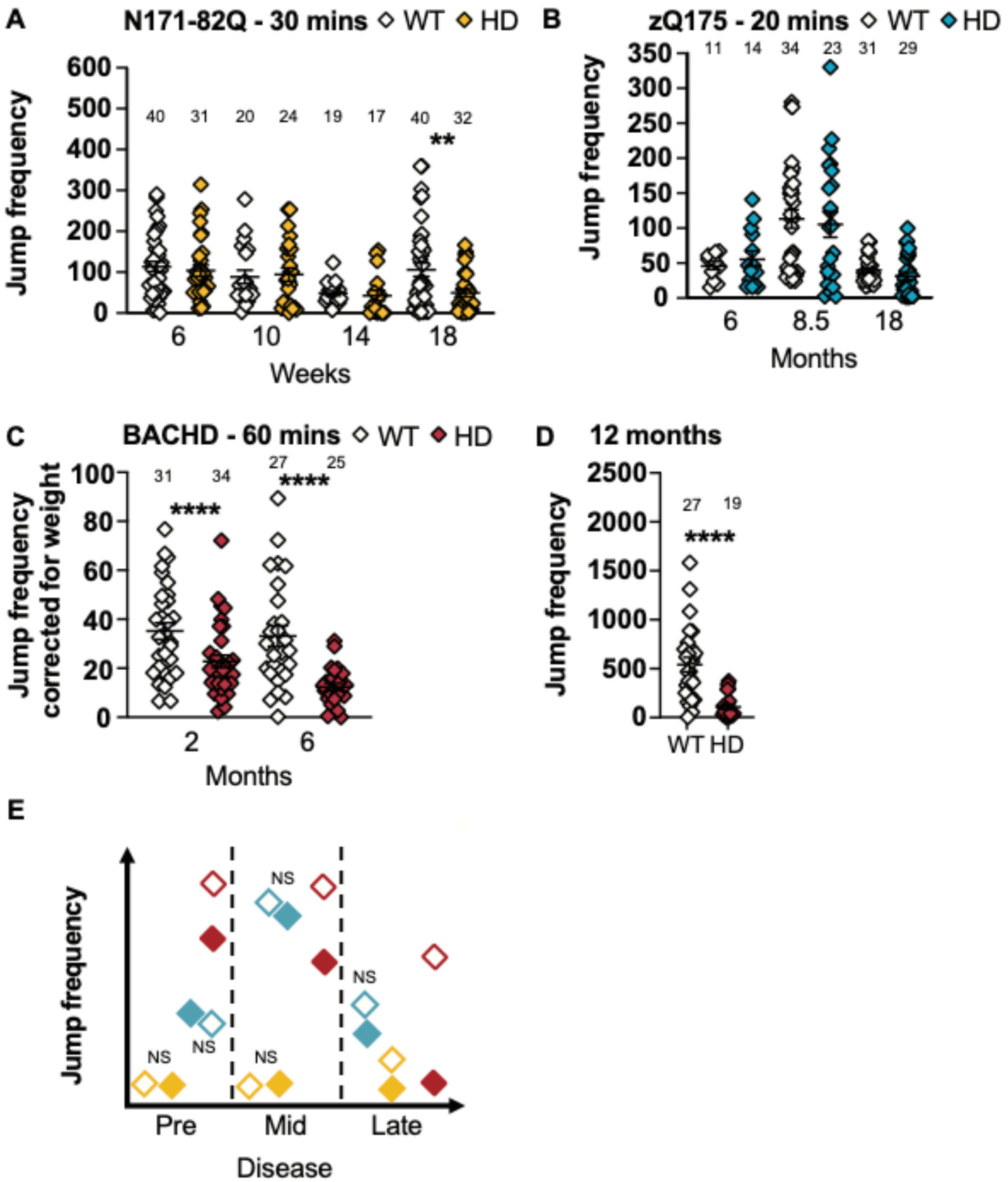

Supplementary Figure 8. Two HD mice models jump less frequently in the activity chamber. N171-82Q mice jump less frequently in the activity chamber in late disease (*MWU* = 418, p = 0.006) (A). while zQ175 mice jump at the same rate as WT mice (B). BACHD mice jump less frequently than WT mice throughout the disease (2 months: $t = 4.064$, $p < 0.0001$; 6 months: $t = 4.625$, $p < 0.0001$; 12 months: *MWU* = 57, $p < 0.0001$). (C, D). E) Representative model of the jumping frequency in the activity chamber as the disease progresses. All comparisons are made to WT mice of the same background. Sample sizes are presented for each group. HD, Huntington's disease carrier; NS, non-significant; WT, wild-type. Data represent mean ± SEM. **$p \leq 0.01$ and ****$p \leq 0.0001$ indicate a significant difference by a Student's t-test or Mann-Whitney test for each time point.

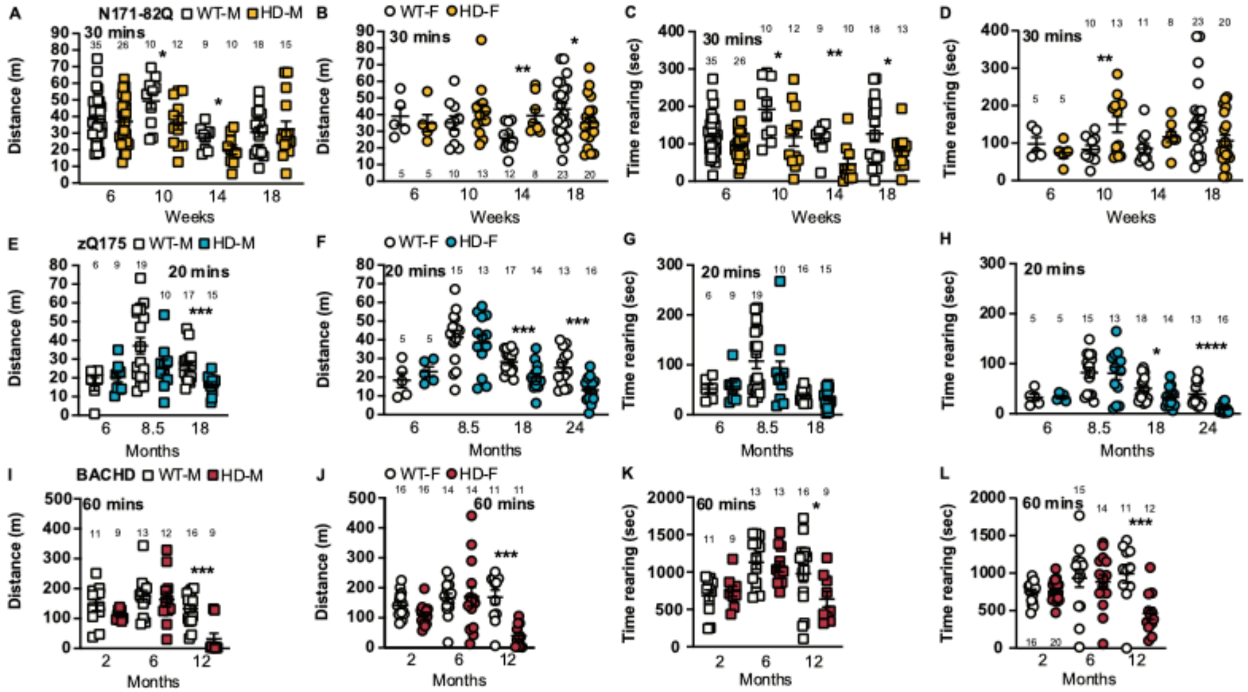

Supplementary Figure 9. HD mouse models of both sexes separately generally travel shorter distances and spend less time rearing in the activity chamber. A) N171-82Q males travel a shorter distance in the activity chamber in mid-disease (10 weeks: $t = 2.096$, $p = 0.02$) and late disease (14 weeks: $t = 2.021$, $p = 0.03$). B) In contrast, N171-82Q females travel a greater distance in mid-disease (14 weeks: $t = 3.146$, $p = 0.003$) and a shorter distance in late-disease (18 weeks: $t = 1.703$, $p = 0.05$). C) N171-82Q males spend less time rearing in mid- ($t = 2.150$, $p = 0.02$) to late-disease (14 weeks: *MWU* = 14, $p = 0.005$; 18 weeks: $t_w = 1.899$, $p = 0.03$). D) N171-82Q females present an increase in the time spent rearing in mid-disease ($t = 2.813$, $p = 0.006$). zQ175 male and female mice travel a shorter distance in the activity chamber in late disease (Male – 18 months: $t_w = 4.176$, $p = 0.0002$; Female – 18 months: $t = 3.505$, $p = 0.0008$; 24 months: $t = 3.922$, $p = 0.0003$) (E, F). zQ175 males spend a similar time rearing throughout the disease when compared to WT males (G) while females present a decrease in late-disease (18 months: $t = 2.212$, $p = 0.02$; 24 months: *MWU* = 18.5, $p < 0.0001$) (H). BACHD males and females travel a shorter distance in the activity chamber in late-disease only (Male: *MWU* = 16, $p = 0.0004$; Female: $t_w = 4.884$, $p = 0.0001$) (I, J). BACHD males and females also rear for a shorter amount of time in late-disease (Male: $t = 1.993$, $p = 0.03$; Female: $t = 3.720$, $p = 0.0006$) (K, L). All comparisons are made to WT mice of the same background. Sample sizes are presented for each group. HD, Huntington's disease carrier; F, female; M, male; WT, wild-type. Data represent mean ± SEM. $*p \leq 0.05$, $**p \leq 0.01$, $***p \leq 0.001$, and $****p \leq 0.0001$ indicate a significant difference by a Student's t-test with or without Welch's correction or Mann-Whitney test for each time point.

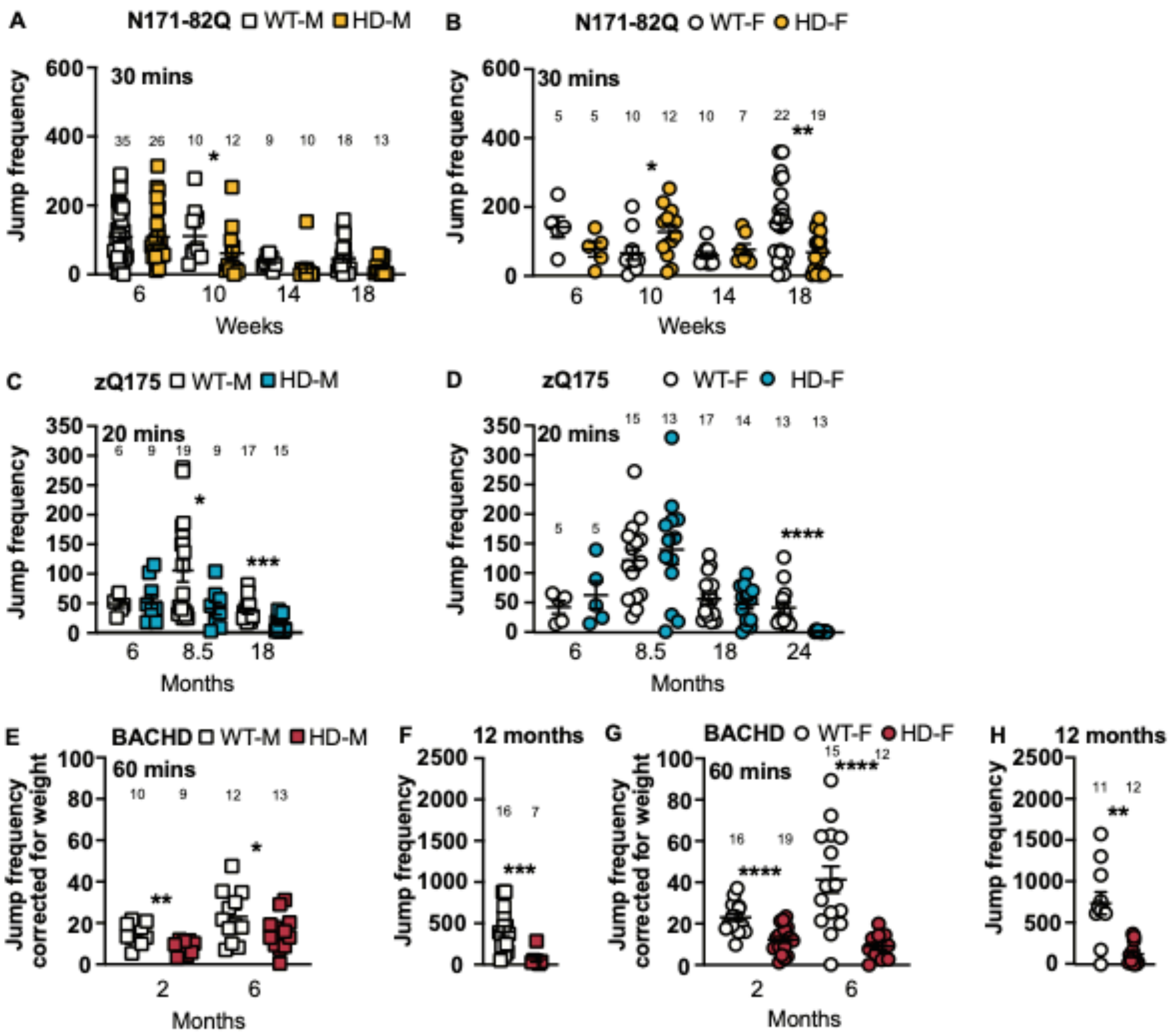

Supplementary Figure 10. HD mouse models of both sexes generally jump less frequently in the activity chamber. A) N171-82Q males jump less frequently in the activity chamber in mid-disease (*MWU* = 32, $p = 0.03$). B) N171-82Q females initially jump more frequently in mid-disease (*MWU* = 31, $p = 0.03$) followed by a significant decrease in late-disease (18 weeks: $t = 3.154$, $p = 0.002$). zQ175 males and females jump less frequently in the activity chamber in mid- to late-disease (Male – 8.5 months: *MWU* = 47.5, $p = 0.03$; 18 months: *MWU* = 45, $p = 0.0006$; Female - 24 months: *MWU* = 0, $p < 0.0001$) (C, D). BACHD males and females jump less frequently in the activity chamber throughout the disease (Males: 2 months: $t = 3.032$, $p = 0.004$; 6 months: $t = 1.817$, $p = 0.04$; 12 months: *MWU* = 7, $p = 0.0002$; Females: 2 months: $t = 4.708$, $p < 0.0001$; 6 months: $t_w = 4.873$, $p < 0.0001$; 12 months: $t_w = 4.322$, $p = 0.001$) (E-H). All comparisons are made to WT mice of the same background. Sample sizes are presented for each group. HD, Huntington's disease carrier; F, female; M, male; WT, wild-type. Data represent mean ± SEM. *$p \leq 0.05$, **$p \leq 0.01$, ***$p \leq 0.001$, and ****$p \leq 0.0001$ indicate a significant difference by a Student's t-test with or without Welch's correction or Mann-Whitney test for each time point.

Supplementary Table 1. Recommended sample sizes to detect a therapeutic benefit for different behavioral tests in males of three HD mouse models

**Mouse model**	**N171-82Q**				**zQ175**			**BACHD**		
Disease stage	Early	Mid	Late	Late	Early	Mid	Late	Early	Mid	Late
Age (Unit)	6	10	14	18	6	8.5	18	2	6	12
Measure	Weeks				Months			Months		
Weight 25%<br>50%<br>75%<br>Other	ND	ND<br>51 / grp<br>23 / grp<br>**95%: 14 / grp**	**9 / grp**<br>**<6 / grp**<br>**<6 / grp**	25 / grp<br>**7 / grp**<br>**<6 / grp**	ND<br>41 / grp<br>**18 / grp**<br>**85%: 14 / grp**	ND<br>ND<br>29 / grp<br>**100%: 17 / grp**	36 / grp<br>**9 / grp**<br>**<6 / grp**	ND<br>33 / grp<br>**15 / grp**	ND<br>58 / grp<br>26 / grp<br>**100%: 15 / grp**	ND
Rotarod Performance 25%<br>50%<br>75%<br>Other	ND<br>33 / grp<br>**15 / grp**	ND	**6 / grp**<br>**<6 / grp**<br>**<6 / grp**	**<6 / grp**<br>**<6 / grp**<br>**<6 / grp**	ND	ND<br>ND<br>30 / grp<br>**100%: 17 / grp**	ND	ND<br>**16 / grp**<br>**8 / grp**	56 / grp<br>**14 /grp**<br>**7 /grp**	ND
FGS 25%<br>50%<br>75%<br>Other	ND<br>58 / grp<br>26 / grp<br>**100%: 15 /grp**	ND<br>49 / grp<br>**19 / grp**<br>**85%: 15 / grp**	ND<br>58 / grp<br>26 / grp<br>**100%: 15 / grp**	**15 / grp**<br>**<6 / grp**<br>**<6 / grp**	ND<br>63 / grp<br>28 / grp<br>**100%: 16 / grp**	NA	NA	ND<br>28 / grp*<br>**13 / grp***	ND<br>52 / grp*<br>24 / grp*<br>**100%: 13 / grp***	ND
DR – T-turn 25%<br>50%<br>75%<br>Other	ND	ND	ND	ND	ND<br>ND<br>37 / grp<br>100%: 21 / grp	NA	NA	ND	ND	**8 / grp**<br>**<6 / grp**<br>**<6 / grp**
DR – T-total 25%<br>50%<br>75%<br>Other	ND<br>24 / grp<br>**11 /grp**	ND	ND<br>ND<br>51 / grp<br>100%: 29 / grp	ND	ND<br>60 / grp<br>27 /grp<br>**100%: 15 /grp**	NA	NA	**9 / grp**<br>**<6 / grp**<br>**<6 / grp**	ND<br>28 / grp<br>**13 /grp**	**14 / grp**<br>**<6 /grp**<br>**<6 /grp**
DR – Latency 25%<br>50%<br>75%<br>Other	**<6 / grp**<br>**<6 / grp**<br>**<6 / grp**	ND	ND	ND	ND	NA	NA	ND	ND	**18 / grp**<br>**<6 /grp**<br>**<6 /grp**
NB – Cross time 25%<br>50%<br>75%<br>Other	ND	NA	NA	NA	NA	NA	NA	**17 / grp**<br>**<6 / grp**<br>**<6 / grp**	26 / grp<br>**7 / grp**<br>**<6 / grp**	30 / grp<br>**8 / grp**<br>**<6 / grp**
NB – Slips 25%<br>50%<br>75%<br>Other	ND<br>ND<br>41 / grp<br>100%: 23 / grp	NA	NA	NA	NA	NA	NA	ND	ND<br>ND<br>32 / grp<br>**100%: 18 / grp**	ND
NB – Latency 25%<br>50%<br>75%<br>Other	ND<br>**19 / grp**<br>**9 / grp**	NA	NA	NA	NA	NA	NA	ND	ND	ND
CT – Frequency 25%<br>50%<br>75%<br>Other	NA	NA	NA	NA	NA	NA	NA	ND<br>**17 / grp***<br>**8 / grp***	**4 months old**<br>ND<br>40 / grp*<br>100%: 23 / grp*	ND
AC – Distance 25%<br>50%<br>75%<br>Other	ND	ND<br>59 / grp<br>26 / grp<br>**100%: 15 / grp**	ND<br>ND<br>**15 / grp**<br>**100%: 9 / grp**	ND	ND	ND	41 / grp<br>**11 / grp**<br>**5 / grp**	ND	ND	61 / grp<br>**16 / grp**<br>**7 / grp**
AC – Jump 25%<br>50%<br>75%<br>Other	ND	ND<br>ND<br>50 / grp<br>100%: 28 / grp	ND	ND	ND	48 / grp<br>**12 / grp**<br>**6 / grp**	ND<br>21 / grp<br>**10 /grp**	57 / grp*<br>**15 / grp***<br>**7 / grp***	ND	**17 / grp**<br>**<6 / grp**<br>**<6 / grp**
AC – Rearing 25%<br>50%<br>75%<br>Other	ND	ND<br>61 / grp<br>27 / grp<br>**100%: 16 / grp**	ND<br>29 / grp<br>**13 / grp**	ND<br>48 / grp<br>22 / grp<br>**90%: 15 / grp**	ND	ND	ND	ND	ND	ND<br>42 / grp<br>**19 / grp**<br>**85%: 15 / grp**

HD male mice recommended sample sizes to detect a normalization of the behavior by 25, 50, 75% or more. Sample sizes in bold are practical. AC, Activity chamber; CT, Climbing test; DR, Descending rod; FGS, Forelimb grip strength; H, Huntington's disease carrier; NA, not available, NB, Narrow beam; ND, not determined; T-time, Time to descend the descending rod; T-turn, Time to turn downward on the descending rod; W, wild-type of the same background. *Weight-corrected data.

Supplementary Table 2. Recommended sample sizes to detect a therapeutic benefit for different behavioral tests in females of three HD mouse models

**Mouse model**	**N171-82Q**				**zQ175**				**BACHD**		
Disease stage	Early	Mid	Late	Late	Early	Mid	Late	Late	Early	Mid	Late
Age (Unit)	6	10	14	18	6	8.5	18	24	2	6	12
Measure	Weeks				Months				Months		
Weight 25%<br>50%<br>75%<br>Other	ND	ND<br>61 / grp<br>48 / grp<br>100%: 35 / grp	ND<br>32 / grp<br>**14 / grp**	26 / grp<br>**9 / grp**<br>**<6 / grp**	ND<br>ND<br>48 / grp<br>100%: 27 / grp	ND<br>24 / grp<br>**100%: 11 / grp**	30 / grp<br>**8 / grp**<br>**<6 / grp**	ND<br>47 / grp<br>21 / grp<br>**85%: 17 / grp**	ND<br>40 / grp<br>**18 / grp**<br>**85%: 14 / grp**	37 / grp<br>**10 / grp**<br>**<6 / grp**	ND<br>**19 / grp**<br>**9 / grp**
Rotarod Performance 25%<br>50%<br>75%<br>Other	ND	ND<br>61 / grp<br>28 / grp<br>**90%: 20 / grp**	ND	**<6 / grp**<br>**<6 / grp**<br>**<6 / grp**	ND	ND	ND	58 /grp<br>**15 / grp**<br>**7 / grp**	ND<br>35 / grp<br>**16 / grp**<br>**85%: 14 / grp**	ND<br>**17 / grp**<br>**8 / grp**<br>**80%: 7 / grp**	ND<br>**18 / grp**<br>**8 / grp**
FGS 25%<br>50%<br>75%<br>Other	ND<br>34 / grp<br>**16 / grp**<br>**80%: 14 / grp**	35 / grp<br>**9 / grp**<br>**<6 / grp**	**16 / grp**<br>**<6 / grp**<br>**<6 / grp**	**<6 / grp**<br>**<6 / grp**<br>**<6 / grp**	ND<br>41 / grp<br>19 / grp<br>**85%: 15 / grp**	NA	NA	NA	41 / grp*<br>**11 / grp***<br>**<6 / grp***	**13 / grp***<br>**<6 / grp***<br>**<6 / grp***	ND<br>22 / grp*<br>**10 / grp***
DR – T-turn 25%<br>50%<br>75%<br>Other	ND	ND	ND	NA**	ND	NA	NA	NA	**12 / grp**<br>**<6 / grp**<br>**<6 / grp**	ND	50 / grp<br>**13 / grp**<br>**6 / grp**
DR – T-total 25%<br>50%<br>75%<br>Other	ND	ND<br>21 / grp<br>**10 / grp**	ND	NA**	ND<br>ND<br>34 /grp<br>**100%: 19 /grp**	NA	NA	NA	**<6 / grp**<br>**<6 / grp**<br>**<6 / grp**	ND<br>27 / grp<br>**12 /grp**	18 / grp<br>**<6 /grp**<br>**<6 /grp**
DR – Latency 25%<br>50%<br>75%<br>Other	**<6 / grp**<br>**<6 / grp**<br>**<6 / grp**	ND	**<6 / grp**<br>**<6 / grp**<br>**<6 /grp**	NA**	ND	NA	NA	NA	**<6 / grp**<br>**<6 / grp**<br>**<6 / grp**	ND	ND
NB – Cross time 25%<br>50%<br>75%<br>Other	ND<br>25 / grp<br>**11 / grp**	NA	NA	NA	NA	NA	NA	NA	**<6 / grp**<br>**<6 / grp**<br>**<6 / grp**	ND	ND<br>ND<br>34 / grp<br>**100%: 20 / grp**
NB – Slips 25%<br>50%<br>75%<br>Other	ND	NA	NA	NA	NA	NA	NA	NA	ND<br>32 / grp<br>**15 / grp**	ND<br>ND<br>49 / grp<br>100%: 28 / grp	ND<br>ND<br>29 / grp<br>**100%: 16 / grp**
NB – Latency 25%<br>50%<br>75%<br>Other	ND	NA	NA	NA	NA	NA	NA	NA	ND	ND	ND<br>ND<br>36 / grp<br>**100%: 20 / grp**
CT – Frequency 25%<br>50%<br>75%<br>Other	NA	NA	NA	NA	NA	NA	NA	NA	ND<br>43 / grp*<br>**19 / grp***<br>**100%: 11 / grp***	**4 months old**<br>**16 / grp***<br>**8 / grp***	ND<br>**11 / grp***<br>**5 / grp***
AC – Distance 25%<br>50%<br>75%<br>Other	ND	ND	ND<br>37 / grp<br>**17 / grp**<br>**80%: 15 / grp**	ND<br>ND<br>ND<br>100%: 38 /grp	ND	ND	ND<br>37 / grp<br>**17 / grp**<br>**85%: 13 / grp**	60 / grp<br>**15 / grp**<br>**7 / grp**	ND	ND	ND<br>**<6 / grp**<br>**<6 / grp**
AC – Jump 25%<br>50%<br>75%<br>Other	ND	ND<br>ND<br>35 / grp<br>**100%: 20 / grp**	ND	ND<br>23/ grp<br>**10 / grp**	ND	ND	ND	**<6 / grp**<br>**<6 / grp**<br>**<6 / grp**	ND<br>**18 / grp***<br>**8 / grp***	ND<br>**<6 / grp***<br>**<6 / grp***	**10 / grp**<br>**<6 / grp**<br>**<6 / grp**
AC – Rearing 25%<br>50%<br>75%<br>Other	ND	ND<br>ND<br>29 / grp<br>**100%: 17 / grp**	ND	ND	ND	ND	ND<br>58 / grp<br>26 / grp<br>**100%: 15 / grp**	**18 / grp**<br>**<6 / grp**<br>**<6 / grp**	ND	ND	53 / grp<br>**14 / grp**<br>**6 / grp**

HD female mice recommended sample sizes to detect a normalization of the behavior by 25, 50, 75% or more. Sample sizes in bold are practical. AC, Activity chamber; CT, Climbing test; DR, Descending rod; FGS, Forelimb grip strength; H, Huntington's disease carrier; NA, not available, NB, Narrow beam; ND, not determined; T-time, Time to descend the descending rod; T-turn, Time to turn downward on the descending rod; W, wild-type of the same background. *Weight-corrected data.

Supplementary Table 3. Behavioral changes over disease progression in males of three HD mouse models

**Mouse model**	**N171-82Q**				**zQ175**			**BACHD**		
Disease stage	Early	Mid	Late	Late	Early	Mid	Late	Early	Mid	Late
Age (Unit) / Measure	6	10	14	18	6	8.5	18	2	6	12
	Weeks				Months			Months		
Weight	=	↓	↓↓↓↓	↓↓↓↓	↓↓	↓	↓↓↓↓	↑↑↑	↑↑	=
Rotarod Performance	↓↓	=	↓↓↓	↓↓↓↓	=	↓	=	↓↓↓↓	↓↓↓	=
Rotarod Learning	=	=	↓↓↓↓	=	=	↓	=	=	↓↓↓	=
FGS	↓↓	↓↓	↓	↓↓↓↓	↓	NA	NA	↓↓↓*	↓↓↓*	=
DR – T-turn	=	=	=	=	↑	NA	NA	=	=	↓↓↓
DR – T-total	↓↓	=	↑	=	↑	NA	NA	↓↓↓	↓↓	↓↓↓↓
DR – Latency	↓	=	=	=	=	NA	NA	=	=	↓↓
NB – Cross time	=	NA	NA	NA	NA	NA	NA	↓↓↓	↓↓↓	↓↓↓↓
NB – Slips	↑	NA	NA	NA	NA	NA	NA	=	↑	=
NB – Latency	↑↑↑	NA	NA	NA	NA	NA	NA	=	=	=
CT – Frequency	NA	NA	NA	NA	NA	NA	NA	↓↓*	↓*	=
AC – Distance	=	↓	↓	=	=	=	↓↓↓	=	=	↓↓↓
AC – Jump	=	↓	=	=	=	↓	↓↓↓	↓*	↓↓*	↓↓↓
AC – Rearing	=	↓	↓↓	↓	=	=	=	=	=	↓
Recommended	Rotarod<br>FGS<br>*DR*<br>NB	Weight<br>FGS<br>*DR*	Weight<br>Rotarod<br>FGS<br>DR<br>AC	Weight<br>Rotarod<br>FGS<br>AC	Weight<br>FGS<br>DR	Weight<br>Rotarod	Weight<br>AC	*Weight*<br>Rotarod<br>FGS<br>*DR*<br>*NB*<br>CT<br>AC	*Weight*<br>Rotarod<br>FGS<br>*DR*<br>NB<br>CT<br>AC	*DR*<br>*NB*<br>AC

Black arrows represent expected behavior direction while red arrows indicate behavior not following the human HD pathology. Italicized recommended tests indicate unexpected result direction. *Weight-corrected results. AC, Activity chamber; CT, Climbing test; DR, Descending rod; FGS, Forelimb grip strength; NA, not available, NB, Narrow beam; T-time, Time to descend the descending rod; T-turn, Time to turn downward on the descending rod.

Supplementary Table 4. Behavioral changes over disease progression in females of three HD mouse models

**Mouse model**	**N171-82Q**				**zQ175**				**BACHD**		
Disease stage	Early	Mid	Late	Late	Early	Mid	Late	Late	Early	Mid	Late
Age (Unit)	6	10	14	18	6	8.5	18	24	2	6	12
Measure	Weeks				Months				Months		
Weight	=	↓	↓	↓↓↓	↓	↓↓	↓↓↓↓	↓↓	↑↑↑	↑↑↑↑	↑↑↑↑
Rotarod Performance	=	↓↓	=	↓↓↓	=	=	=	↓	↓↓↓	↓↓↓↓	↓↓↓
Rotarod Learning	=	=	↓↓↓	=	=	=	↑	=	↓↓	↓↓↓	=
FGS	↓↓	↓↓↓↓	↓↓↓↓	↓↓↓↓	↓↓	NA	NA	NA	↓↓↓↓*	↓↓↓↓*	↓↓↓*
DR – T-turn	=	=	=	NC	=	NA	NA	NA	↓↓	=	↓↓
DR – T-total	=	↓↓	=	NC	↑	NA	NA	NA	↓↓↓	↓↓	↓↓
DR – Latency	↓↓	=	↓↓	NC	=	NA	NA	NA	↓↓	=	=
NB – Cross time	↓	NA	NA	NA	NA	NA	NA	NA	↓	=	↓
NB – Slips	=	NA	NA	NA	NA	NA	NA	NA	↑↑↑	↑	↑↑↑
NB – Latency	=	NA	NA	NA	NA	NA	NA	NA	=	=	↓
CT – Frequency	NA	NA	NA	NA	NA	NA	NA	NA	↓↓*	↓↓↓*	↓↓*
AC – Distance	=	=	↑↑	↓	=	=	↓↓↓	↓↓↓	=	=	↓↓↓
AC – Jump	=	↑	=	↓↓	=	=	=	↓↓↓↓	↓↓↓↓*	↓↓↓↓*	↓↓
AC – Rearing	=	↑↑	=	=	=	=	↓	↓↓↓↓	=	=	↓↓↓
Recommended	FGS<br>*DR*<br>*NB*	Weight<br>Rotarod<br>FGS<br>*DR*<br>*AC*	Weight<br>Rotarod<br>FGS<br>*DR*<br>*AC*	Weight<br>Rotarod<br>FGS<br>AC	Weight<br>FGS<br>DR	Weight	Weight<br>*Rotarod*<br>AC	Weight<br>Rotarod<br>AC	*Weight*<br>Rotarod<br>FGS<br>NB<br>CT<br>AC	*Weight*<br>Rotarod<br>FGS<br>*DR*<br>NB<br>CT<br>AC	*Weight*<br>Rotarod<br>FGS<br>*DR*<br>NB<br>CT<br>AC

Black arrows represent expected behavior direction while red arrows indicate behavior not following the human HD pathology. Italicized recommended tests indicate unexpected result direction. *Weight-corrected results. AC, Activity chamber; CT, Climbing test; DR, Descending rod; FGS, Forelimb grip strength; NA, not available, NB, Narrow beam; NC, Mice were not capable of performing the task; T-time, Time to descend the descending rod; T-turn, Time to turn downward on the descending rod.
